# CELSR3 mRNA expression is increased in hepatocellular carcinoma and indicates poor prognosis

**DOI:** 10.7717/peerj.7816

**Published:** 2019-10-07

**Authors:** Xuefeng Gu, Hongbo Li, Ling Sha, Yuan Mao, Chuanbing Shi, Wei Zhao

**Affiliations:** 1 Medical School, Southeast University, Nanjing, Jiangsu, China; 2 The Second Hospital of Nanjing, Medical School, Southeast University, Nanjing, Jiangsu, China; 3 Department of Hepatology, Infectious diseases Hospital Affliated to Soochow University, Suzhou, Jiangsu, China; 4 Department of Neurology, Affiliated Drum Tower Hospital of Nanjing University Medical School, Nanjing, Jiangsu, China; 5 Department of Hematology and Oncology, Geriatric Hospital of Nanjing Medical University, Jiangsu Province Geriatric Hospital, Nanjing, Jiangsu, China; 6 Department of Pathology, Pukou District Central Hospital, Pukou Branch of Jiangsu Province Hospital, The First Affiliated Hospital of Nanjing Medical University, Nanjing, Jiangsu, China

**Keywords:** GEO, The Cancer Genome Atlas, GSEA, HCC, CELSR3, CCLE

## Abstract

**Objective:**

Hepatocellular carcinoma (HCC) is a disease that is associated with high mortality; currently, there is no curative and reliable treatment. Cadherin EGF LAG seven-pass G-type receptor 3 (CELSR3) is the key signaling molecule in the wingless and INT-1/planar cell polarity (WNT/PCP) pathway. This study aimed to elucidate the prognostic significance of CELSR3 in HCC patients.

**Methods:**

The Cancer Genome Atlas (TCGA) database, the Cancer Cell Line Encyclopedia (CCLE) database and the Gene Expression Omnibus (GEO) database were used to analyze the expression of CELSR3 mRNA in HCC samples and cells. The relationship between CELSR3 mRNA and clinical features was assessed by the chi-square test. the diagnostic and predictive value of CELSR3 mRNA expression were analyzed using the receiver operating characteristic (ROC) curve. Kaplan–Meier curve and Cox regression analyses were performed to assess the prognostic value of CELSR3 mRNA in HCC patients. Finally, all three cohorts database was used for gene set enrichment analysis(GSEA) and the identification of CELSR3-related signal transduction pathways.

**Results:**

The expression of CELSR3 mRNA was upregulated in HCC, and its expression was correlated with age (*P* = 0.025), tumor status (*P* = 0.022), clinical stage (*P* = 0.003), T classification (P = 0.010), vital status (*P* = 0.001), and relapse (*P* = 0.005). The ROC curve assessment indicated that CELSR3 mRNA expression has high diagnostic value in HCC and in the subgroup analysis of stage. In addition, the Kaplan-Meier curve and Cox analyses suggested that patients with high CELSR3 mRNA expression have a poor prognosis, indicating that CELSR3 mRNA is an independent prognostic factor for the overall survival of HCC patients. GSEA showed that GO somatic diversification of immune receptors, GO endonuclease activity, GO DNA repair complex and GO somatic cell DNA recombination, were differentially enriched in the meta-GEO cohort, the HCC cell line cohort and the TCGA cohort of the high CELSR3 mRNA expression phenotype.

**Conclusion:**

Our results indicate that CELSR3 mRNA is involved in the progression of cancer and can be used as a biomarker for the prognosis of HCC patients.

## Introduction

Hepatocellular carcinoma (HCC) is currently one of the most common malignant tumors worldwide. Globally, the incidence rate is ranked 6th, and the mortality rate is ranked 4th among malignant tumors. There were approximately 840,000 newly diagnosed patients worldwide in 2018, and approximately 780,000 people die annually (Bray et al., 2018). Although the current treatment methods for HCC have made great progress, strategies for HCC treatment are still limited. Available studies have indicated that some genes are closely related to the prognosis of HCC and thus might be used as valuable biomarkers for the treatment of this disease (Petrizzo et al., 2018). Therefore, it is of great importance to search for markers of progression and poor prognosis of HCC.

As a calcium-dependent transmembrane glycoprotein, cadherin is characterized by an extracellular calcium-binding domain (composed of a sequence of approximately 110 repeated amino acids), and it plays important roles in embryonic development and synaptogenesis (Gumbiner, 1996; Takeichi, 1991; Halbleib & Nelson, 2006; Takeichi, 2007). The cadherin EGF LAG seven-pass G-type receptor (Celsr) gene family is related to nonclustered protocadherin (Wu & Maniatis, 2000). In mammals, evolution of the cadherin gene family led to three genes, CELSR1, Celsr2, and CELSR3 (Beall, Boekelheide & Johnson, 2005; Formstone & Little, 2001; Tissir et al., 2002; Formstone & Mason, 2005). CELSR3 is the key signaling molecule in the wingless and INT-1/planar cell polarity (WNT/PCP) pathway, an important pathway that controls the polarity of tissues and cell migration (Katoh, 2005). Recent studies (Katoh & Katoh, 2007) have shown that CELSR3 expression in adult brain tumors reflects the role of CELSR3 in carcinogenic processes. CELSR3 is selectively upregulated in pancreatic stellate cells (PSCs) of pancreatic tumors (Erkan et al., 2010). Asad et al. (2014) found that CELSR3 is highly expressed in ovarian cancer. Pan et al. (2019) constructed a ceRNA network in head and neck squamous cell carcinoma (HNSCC), and CELSR3 (as a differential gene of the ceRNA network) indicated a worse prognosis in the overall survival of HNSCC. Xu et al. (2019) compared normal cervical tissues and human papillomavirus (HPV) positive cervical cancer tissues and identified CELSR3 as a novel candidate gene related to the progression and carcinogenicity of cervical lesions. To determine the molecular basis of colorectal cancer (CRC) metastasis, Goryca et al. performed a whole-exome and genome-scale transcriptome sequencing of seven liver metastases and their matched primary tumors and normal tissues. The authors found that CELSR3 had exclusive metastatic variants (EMV) in four patients (Goryca et al., 2018).

However, there is still a lack of research on the prognostic value of CELSR3 in HCC. whether CELSR3 might also be a specific marker in liver tumors remains to be elucidated.

In this study, we evaluated the expression of CELSR3 mRNA in HCC, analyzed the relationship between CELSR3 mRNA expression and clinical features, and investigated the prognostic significance of CELSR3 mRNA in HCC patients. Finally, gene set enrichment analysis (GSEA) was performed to further explore the biological pathways by which CELSR3 participates in HCC pathogenesis.

This study demonstrates, for the first time, that CELSR3 is a prognostic gene of HCC and may represent a new potential marker associated with HCC progression. Furthermore, it is correlated with G1 pathway, ATRBRCA pathway, E2F targets, G2 M checkpoint and spermatogenesis. GO somatic diversification of immune receptors, GO endonuclease activity, GO DNA repair complex and GO somatic cell DNA recombination may be important biological pathways through which CELSR3 mRNA participates in the pathogenesis of liver cancer, which deserve further study.

## Materials and Methods

### Data mining and collection

HCC patients in the TCGA and GEO cohorts meeting the following criteria were included in the study: (1) primary HCC samples (including hepatocellular carcinoma, hepatocholangiocarcinoma (mixed) and fibrolamellar carcinoma); (2) complete RNA-seq data. The exclusion criteria was samples that did not contain enough data for analysis.The gene expression data (423 cases, Workflow Type: HTSeq-Counts) and corresponding clinical information were obtained from The Cancer Genome Atlas (TCGA) Liver Hepatocellular Carcinoma (TCGA-LIHC) study of the official TCGA website (https://cancergenome.nih.gov/).The complete clinical data of the corresponding patients were obtained from cBioPortal (http://www.cbioportal.org/). A total of 373 HCC tissue specimens and 50 adjacent nontumor tissue specimens of HCC were included in the study. The Homo_sapiens.GRCh38.84.chr.gtf.gz file was downloaded from the Ensembl website (https://asia.ensembl.org/index.html), and ID conversion was performed using Perl (version 5.26.1). TCGA original HTSeq-Count data were processed using the Trimmed Mean of M-values (TMM) method for homogenization with edgeR. When an RNA had duplicate data, the average RNA expression was used. Microarray data were downloaded from the Gene Expression Omnibus (GEO) database. The GSE54236 original Series Matrix dataset with survival data was based on the GPL6480 (Agilent-014850 Whole Human Genome Microarray 4x44K G4112F, Agilent Technologies, CA, Palo Alto) (submission date: Jan 21, 2014). The GSE60502 (submission date: Aug 18, 2014) datasets were based on GPL570 (Affymetrix Human Genome U133A Array Affymetrix, Inc, CA,Santa Clara). GSE41804 (submission date: Oct 24, 2012), GSE45436 (submission date: Mar 22, 2013), GSE62232 (submission date: Oct 09, 2014), and GSE6764 (submission date: Jan 17, 2007) datasets, which were based on GPL570 data (Affymetrix Human Genome U133 Plus 2.0 Array, Affymetrix, Inc, CA, Santa Clara). For the CEL expression profiles, Robust Multiarray Average (RMA) normalization was performed using the affy package. The expression level of CELSR3 mRNA was converted using the log2 value for further analysis. The raw data in the dataset were annotated to obtain the gene expression levels and the average expression values of probes were considered as the expression values of the corresponding genes. We combined the HCC microarray GSE60502 and GSE62232 in the GEO database using Perl (version 5.26.1) and performed batch normalization using the sva package of R. After combination and normalization, 99 HCC patients were divided into high and low expression groups according to the median value of CELSR3 expression for GSEA detection and verification. RNA expression (RNA-Seq) data for CELSR3 in established HCC cell lines were accessed on 01∕02∕19 (*n* = 25) from the Cancer Cell Line Encyclopedia (CCLE) database (https://portals.broadinstitute.org/ccle/about); RNA expression values were reported in reads per kilobase of transcript per million mapped reads (RPKM). We divided the expression of CELSR3 in the HCC cell lines into high and low expression groups using the median value, and then performed GSEA verification to observe the enrichment of CELSR3 differential expression in the HCC cell lines.The GSEA results were presented in the form of multipleGSEA using the Plyr, grid, gridExtra and ggplot2 packages of R.

### Gene set enrichment analysis

GSEA is a computational method that determines whether an a priori defined set of genes shows statistically significant, concordant differences between two biological states (Subramanian et al., 2005). In this study, HCC samples from TCGA data were divided into the CELSR3 high mRNA expression group and the low CELSR3 mRNA expression group based on the median value of CELSR3 mRNA expression. GSEA3.0 was adopted for GSEA. The functional gene set files “c2.cp.biocarta.v6.2.symbols.gmt”, “h.all.v6.2.symbols.gmt” and “c5.all.v6.2.symbols.gmt” were used to summarize and elucidate specific and well-defined biological states or processes. The number of substitutions per analysis was set at 1,000, and gene sets with *P* < 0.05 and a false discovery rate (FDR) < 0.25 were recognized as a significantly enriched.

### Statistical analysis

SPSS statistical software, version 19.0 (SPSS Inc., Chicago, IL, USA) and STATA 12.0 (Stata Corporation, College Station, TX, USA) were used for statistical analysis. The ggplot2 and pROC packages in the statistical software R (R Core Team, 2018) were used for graph generation. Discrete variables are expressed using a box plot to measure expression differences. The chi-square test was used to analyze the relationship between CELSR3 mRNA expression and clinical data. The Kaplan–Meier curve showed that clinicopathologic characteristics were associated with overall and relapse-free survival. Univariate Cox analysis was used to select relevant variables, and subsequently, multivariate Cox analysis was used for prognostic analysis of CELSR3 mRNA expression with regard to the overall and relapse-free survival (RFS) rate of HCC patients. The cutoff value was determined by the median value of CELSR3 mRNA expression. *P* < 0.05 was considered statistically significant.

**Table 1:** Clinical characteristics of the included patients.

**Characteristics**	**Number of sample size (%)**
Age (years)	
≥60	203 (54.42)
<60	169 (45.31)
NA	1 (0.27)
Gender	
Female	121 (32.44)
Male	252 (67.56)
Family history	
Yes	112 (30.03)
No	209 (56.03)
NA	52 (13.94)
Tumor status	
With tumor	112 (30.03)
Tumor free	234 (62.73)
NA	27 (7.24)
Radiation therapy	
No	341 (91.42)
Yes	9 (2.41)
NA	23 (6.17)
Histologic grade	
Grade 1	55 (14.75)
Grade 2	178 (47.72)
Grade 3	123 (32.98)
Grade 4	12 (3.22)
NA	5 (1.34)
Vascular invasion	
Macro	16 (4.29)
Micro	94 (25.20)
None	207 (55.50)
NA	56 (15.01)
TNM Stage	
Stage I	172 (46.11)
Stage II	87 (23.32)
Stage III	85 (22.79)
Stage IV	5 (1.34)
NA	24 (6.43)
T classification	
T1	182 (48.79)
T2	95 (25.47)
T3	80 (21.45)
T4	13 (3.49)
TX	1 (0.27)
NA	2 (0.54)
N classification	
N0	253 (67.83)
N1	4 (1.07)
NX	115 (30.83)
NA	1 (0.27)
M classification	
M0	267 (71.58)
M1	4 (1.07)
MX	102 (27.35)
Residual tumor	
R0	326 (87.40)
R1	17 (4.56)
R2	1 (0.27)
RX	22 (5.90)
NA	7 (1.88)
Vital status	
Living	243 (65.15)
Deceased	130 (34.85)
Relapse	
Yes	176 (47.18)
No	146 (39.14)
NA	51 (13.67)

**Notes.**

NA not available T tumor stage N node M metastasis

## Results

### Patient characteristics

The clinical and gene expression data for the 373 cases of primary HCC were downloaded from TCGA. As shown in Table 1, the median age at diagnosis was 61 years. In our study cohort, after excluding data that is not available, 14.9% of the tumors were well differentiated, 48.4% were moderately differentiated, and 36.7% were poorly or not differentiated. The tumor status included 234 cases without tumors (67.6%) and 112 cases with tumors (32.4%). There were 172 cases (49.3%) of stage I primary HCC, 87 cases (24.9%) of stage II HCC, 85 cases (24.4%) of stage III HCC, and 5 cases (1.4%) of stage IV HCC. There were 326 cases without and 18 with residual tumors. Of the subjects who were alive at the last follow-up, the median survival time was 19.42 months (range: 0-120.73 months).

**Figure 1:**
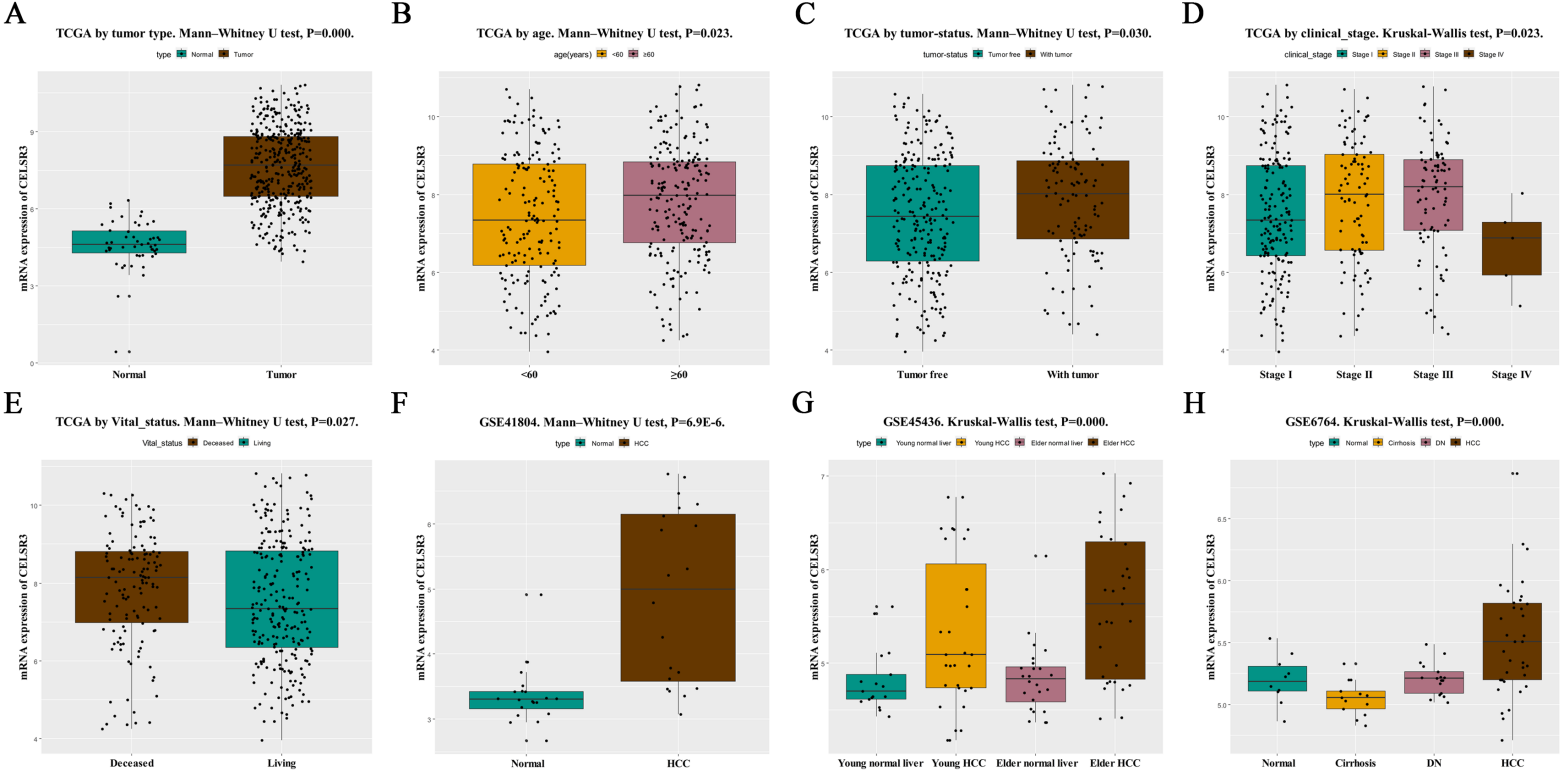
The relationship between CELSR3 mRNA expression and clinical features. Including (A) type, (B) age, (C) tumor status, (D) clinical stage, (E) vital status. The CELSR3 expression of HCC is compared with that in normal, elder/young, and cirrhosis/dysplastic nodule changes based on microarray GSE41804 (F), GSE45436 (G), and GSE6764 (H). CELSR3, Cadherin EGF LAG seven-pass G-type receptor 3; TCGA, The Cancer Genome Atlas; HCC, hepatocellular carcinoma; DN, dysplastic nodule.

**Figure 2:**
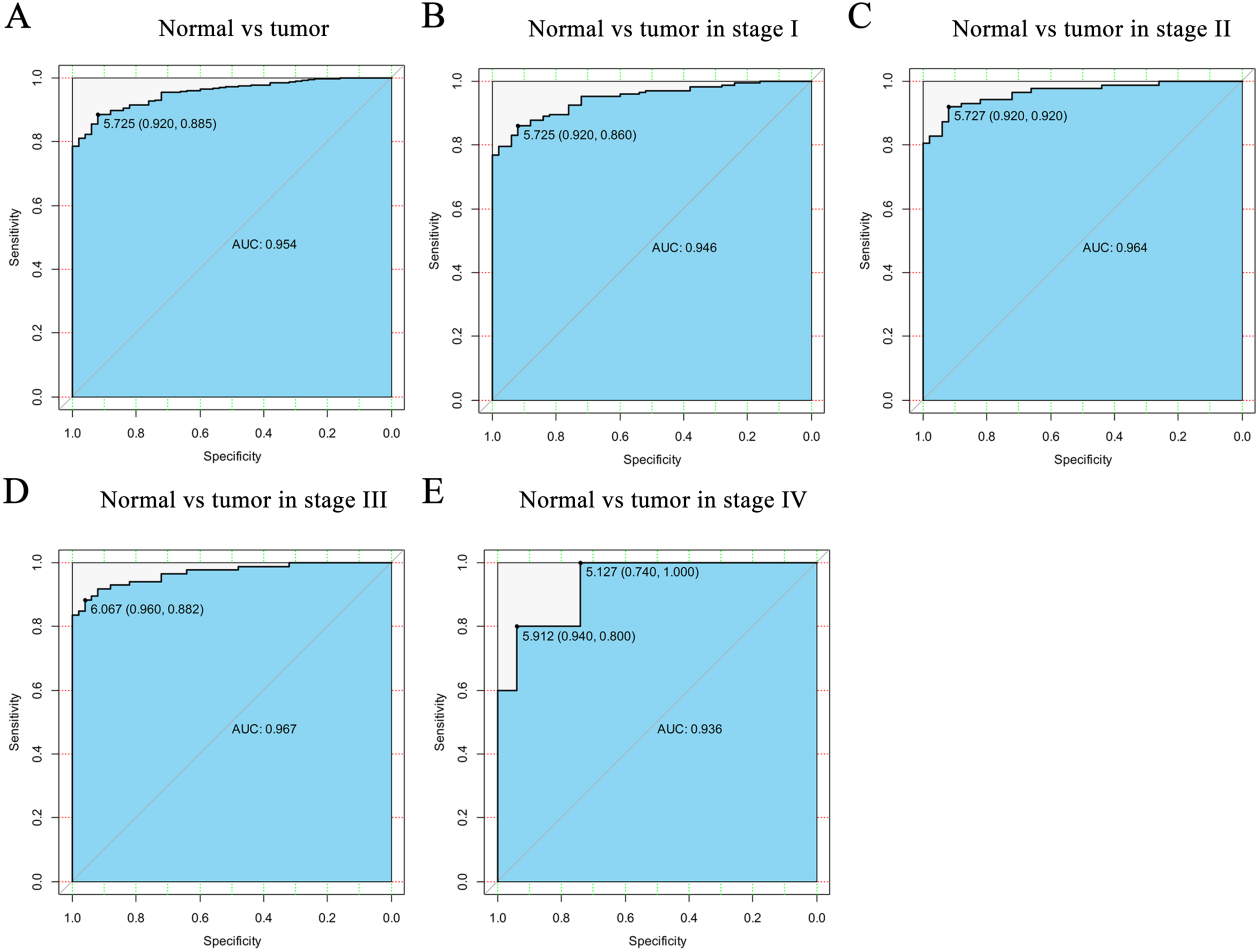
The ROC curve of CELSR3 in LIHC cohort. (A) Nontumor sample and tumor sample. (B) Nontumor sample and tumor sample of stage I. (C) Nontumor sample and tumor sample of stage II. (D) Nontumor sample and tumor sample of stage III. (E) Nontumor sample and tumor sample of stage IV. AUC, area under the curve; ROC, receiver-operating characteristic curve; LIHC, liver hepatocellular carcinoma.

### High CELSR3 mRNA expression in HCC

HCC tissues and normal control tissues in TCGA data were compared using a box plot. As shown in Fig. 1A, the expression of CELSR3 mRNA was higher in HCC tissues (*P* = 0.000). Moreover, there were also different CELSR3 expression levels in the groups classified according to age (Fig. 1B, *P* = 0.023), tumor status (Fig. 1C, *P* = 0.030), stage (Fig. 1D, *P* = 0.023), and vital status (Fig. 1E, *P* = 0.027). The results indicated that CELSR3 is highly expressed in HCC tissues, and the expression of CELSR3 was high in older patients, in those with advanced tumor status or TNM stage III, and in those who had died. It is interesting to note that the expression of CELSR3 decreases in stage IV. When the GEO data were validated, CELSR3 mRNA expression differed for different types of liver tissues in the GSE41804 (Fig. 1F, *P* = 0.000), GSE45436 (Fig. 1G, *P* = 0.000), and GSE6764 (Fig. 1H, *P* = 0.000) datasets. It is worth noting that the expression of CELSR3 mRNA in liver cirrhosis tissues was lower than in normal and HCC tissues.

### Diagnostic function of CELSR3 mRNA

To verify the diagnostic value of CELSR3 in HCC, the ROC curve was used to analyze the AUC of CELSR3 expression associated with clinical pathological parameters of different HCC patients. As shown in Fig. 2A, CELSR3 could effectively distinguish normal liver tissue from HCC tissue. In the receiver operating characteristic (ROC) curve analysis of CELSR3, the area under the curve (AUC) was 0.954, indicating high diagnostic and predictive values of CELSR3. Furthermore, we performed ROC curve analysis of CELSR3 expression in different TNM stage subgroups of HCC patients; our results indicate that high expression of CELSR3 may be a promising diagnostic marker. In the subgroup analysis of stage, the diagnostic values at different stages were also high (AUC: stage *I* = 0.946; stage II = 0.964; stage III = 0.967; stage IV = 0.936; Figs. 2B–2E).

### Relationship between CELSR3 mRNA expression and clinical features of HCC

To validate the relationship between CELSR3 expression and clinical pathological features in HCC patients, we further analyzed the expression level of CELSR3 in HCC patients at different clinical stages. As shown in Table 2, the relationship between CELSR3 mRNA expression and clinical features indicated that CELSR3 mRNA expression was significantly correlated with age (*P* = 0.025), tumor status (*P* = 0.022), clinical stage (*P* = 0.003), T classification (*P* = 0.010), vital status (*P* = 0.001), and relapse (*P* = 0.005). Collectively, these data indicate that the expression of CELSR3 is associated with various important clinical pathological features of HCC.

**Table 2:** Correlation between the clinicopathologic variables and CELSR3 mRNA expression in liver cancer.

**Parameters**	**Groups**	**N**	**CELSR3 expression**				*χ*^2^	*P* value
			**High**	**%**	**Low**	**%**		
Age (years)	≥60	203	110	49.77	93	61.59	5.052	0.025^*^
	<60	169	111	50.23	58	38.41		
Gender	Female	121	57	30.48	64	34.41	0.656	0.418
	Male	252	130	69.52	122	65.59		
Family history	Yes	112	54	33.96	58	35.80	0.120	0.729
	No	209	105	66.04	104	64.20		
Tumor status	With tumor	112	65	38.24	47	26.70	5.252	0.022^*^
	Tumor free	234	105	61.76	129	73.30		
Radiation therapy	No	341	173	97.74	168	97.11	0.139	0.71
	Yes	9	4	2.26	5	2.89		
Histologic grade	Grade 1	55	24	12.90	31	17.03	2.187	0.534
	Grade 2	178	90	48.39	88	48.35		
	Grade 3	123	67	36.02	56	30.77		
	Grade 4	12	5	2.69	7	3.85		
Vascular invasion	Macro	16	8	5.19	8	4.91	0.136	0.934
	Micro	94	47	30.52	47	28.83		
	None	207	99	64.29	108	66.26		
TNM Stage	Stage I	172	73	40.78	99	58.24	14.317	0.003^*^
	Stage II	87	51	28.49	36	21.18		
	Stage III	85	54	30.17	31	18.24		
	Stage IV	5	1	0.56	4	2.35		
T classification	T1	182	76	40.64	106	57.61	13.367	0.010^*^
	T2	95	55	29.41	40	21.74		
	T3	80	50	26.74	30	16.3		
	T4	13	6	3.21	7	3.80		
	TX	1	0	0.00	1	0.54		
N classification	N0	253	131	70.43	122	65.59	1.025	0.599
	N1	4	2	1.08	2	1.08		
	NX	115	53	28.49	62	33.33		
M classification	M0	267	134	71.66	133	71.51	1.040	0.594
	M1	4	1	0.53	3	1.61		
	MX	102	52	27.81	50	26.88		
Residual tumor	R0	326	165	89.67	161	88.46	1.097	0.778
	R1	17	8	4.35	9	4.95		
	R2	1	0	0.00	1	0.55		
	RX	22	11	5.98	11	6.04		
Vital status	Living	243	107	57.22	136	73.12	10.381	0.001^*^
	Deceased	130	80	42.78	50	26.88		
Relapse	Yes	176	101	62.35	75	46.88	7.774	0.005^*^
	No	146	61	37.65	85	53.12		

**Notes.**

* *P* < 0.05.

CELSR3, Cadherin EGF LAG seven-pass G-type receptor 3.

**Figure 3:**
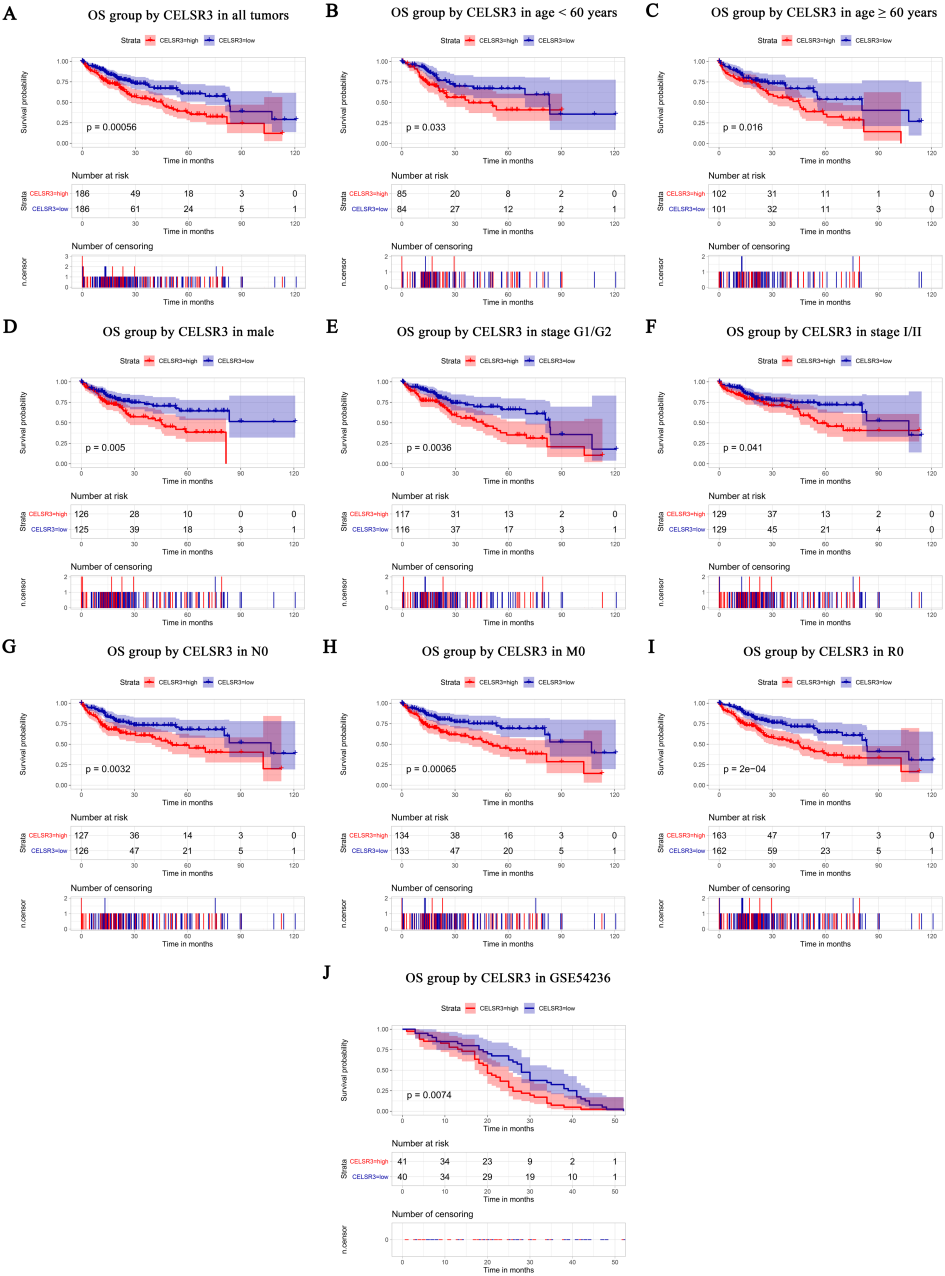
Survival analysis of CELSR3 expression in terms of overall survival (OS). The OS values were analyzed in regards to the mRNA expression level of CELSR3 in all tumors and subgroups of HCC patients. OS analysis of (A) all tumors, (B) age < 60 years, (C) age ≥ 60 years, (D) male, (E) G1 + G2 stage, (F) stage I/II, (G) N0 stage, (H) M0 stage, and (I) R0 stage. Validation group of survival analysis in GSE54236 (J). OS: overall survival; T, tumor stage; N, node; M, metastasis; R, Residual tumor; HCC, hepatocellular carcinoma.

**Figure 4:**
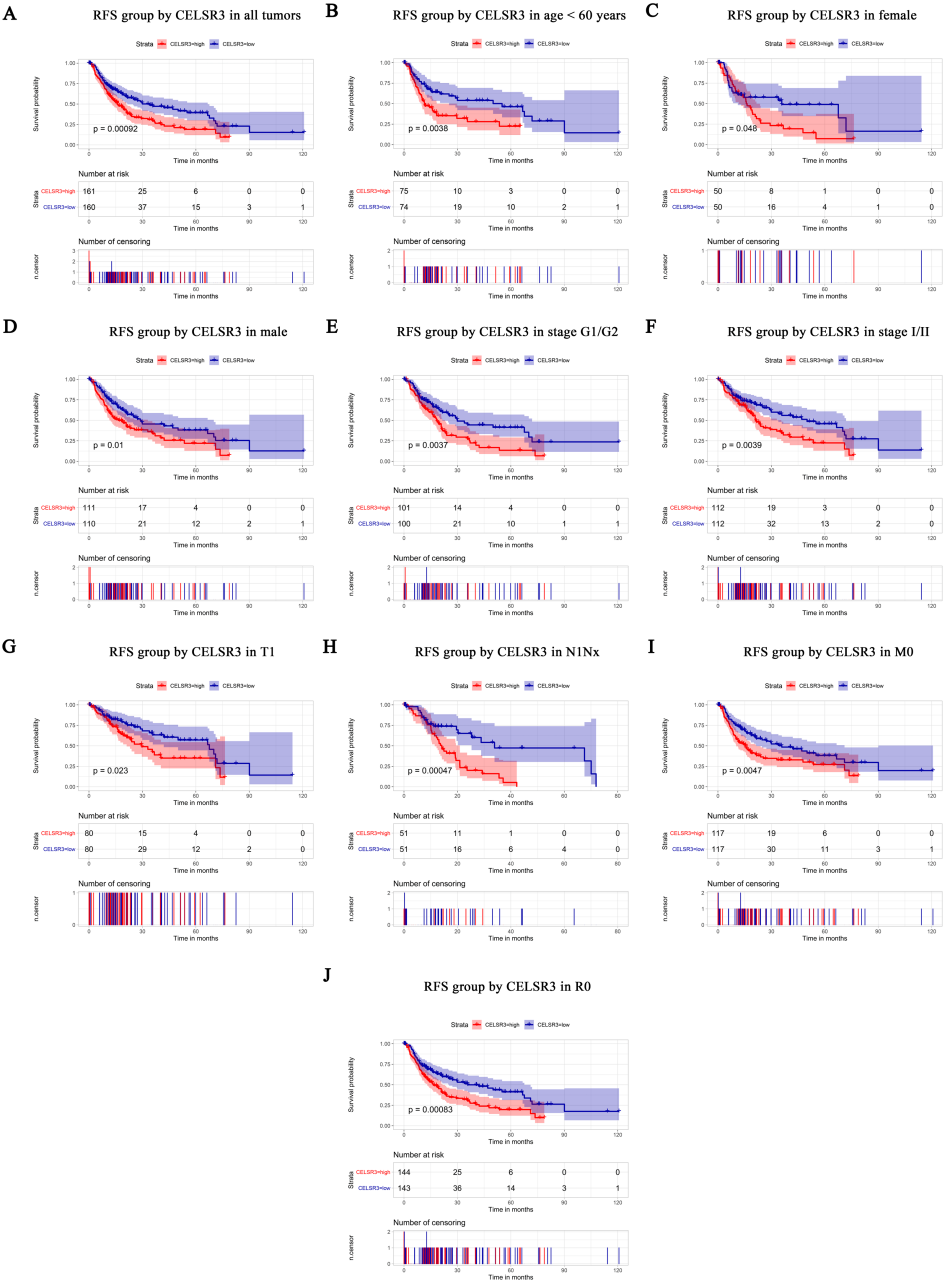
Survival analysis of CELSR3 expression in terms of relapse-free survival (RFS). The RFS values were analyzed in regards to the mRNA expression level of CELSR3 in all tumors and subgroups of HCC patients. RFS analysis of (A) all tumors, (B) age < 60 years, (C) age ≥ 60 years, (D) male, (E) G1 + G2 stage, (F) stage I/II, (G) T1 stage, (H) N1Nx stage, (I) M0 stage, and (J) R0 stage. RFS, relapse-free survival.

### Survival results and multivariate analysis

The Kaplan–Meier survival curve and log-rank test were used to evaluate the relationship between CELSR3 mRNA expression and overall (Fig. 3) and relapse-free survival (Fig. 4). All HCC patients were divided into either the CELSR3 high expression group or CELSR3 low expression group based on the median value of CELSR3 mRNA. We analyzed the OS time of 372 patients. The result showed that the overall survival was poor in patients with high CELSR3 mRNA expression (Fig. 3A; *P* = 0.000). Unexpectedly, RFS analysis produced similar results (Fig. 4A; *P* = 0.000). These results indicated that high CELSR3 expression in patients with HCC can predict a poor prognosis. To further confirm the prognostic value of CELSR3 in patients with HCC, we performed OS and RFS analysis in subgroups of HCC patients. The subgroup analysis indicated that the overall survival was poor in patients with high CELSR3 mRNA expression and an age <60 years or ≥ 60 years, male sex, histological grade G1/G2, stage I/II, N0 stage, M0 stage, and R0 stage (Figs. 3B–3I), and relapse-free survival was poor in the group with high CELSR3 mRNA expression and an age <60 years, female sex or male sex, histological grades G1/G2, stage I/II, T1 stage, N1Nx stage, M0 stage, and R0 stage (Figs. 4B–4J). The survival analysis validated by the GSE54236 data is shown in Fig. 3J (*P* = 0.007). These results demonstrated that high expression of CELSR3 can function as a prognostic biomarker of OS and RFS in subgroups of HCC patients. In different clinical subgroups of HCC patients, the prognostic value of CELSR3 varies, which can guide our clinical practice and deserves further study.

Univariate analysis showed that high expression of CELSR3 mRNA was significantly correlated with poor overall survival and other variables associated with a reduced overall survival rate, including tumor status, stage, T stage, N stage, M stage, and residual tumor. Multivariate analysis using the Cox proportional hazards model indicated that high expression of CELSR3 mRNA (HR = 1.88, *P* = 0.004) and residual tumor (HR = 1.36, *P* = 0.033) were independent prognostic factors for the overall survival of HCC patients (Table 3).

### Identification of CELSR3-related signal transduction pathways by GSEA using TCGA cohort

**Figure 5:**
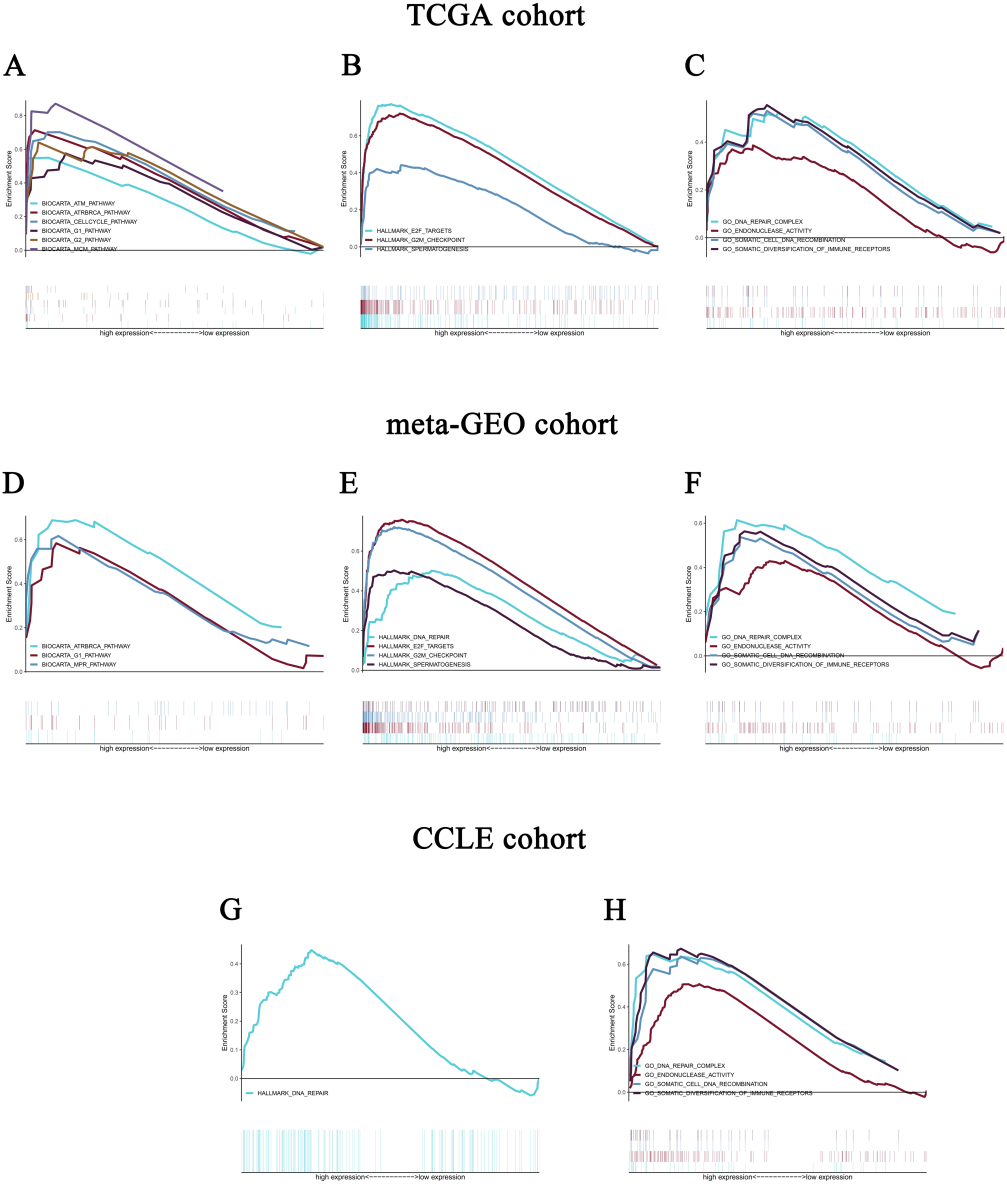
Enrichment plots from gene set enrichment analysis (GSEA). GSEA revealed significant differences in the enrichment of (A) c2.cp.biocarta.v6.2.symbols.gmt, (B) h.all.v6.2.symbols.gmt, and (C) c5.all.v6.2.symbols.gmt in the TCGA cohort. Validation using meta-GEO HCC cohort in the enrichment of (D) c2.cp.biocarta.v6.2.symbols.gmt, (E) h.all.v6.2.symbols.gmt, and (F) c5.all.v6.2.symbols.gmt). Validation using CCLE cohort in the enrichment of (G) h.all.v6.2.symbols.gmt, and (H) c5.all.v6.2.symbols.gmt. GSEA: gene set enrichment analysis; GEO: Gene Expression Omnibus; CCLE: Cancer Cell Line Encyclopedia.

To identify the differentially activated signaling pathways in HCC, gene expression enrichment analysis was performed between datasets with low or high CELSR3 mRNA expression. GSEA revealed significant differences (FDR < 0.25, NOM *P*-value <0.05) in the enrichment of the MSigDB Collection (c2.cp.biocarta.v6.2.symbols.gmt, h.all.v6.2.symbols.gmt, and c5.all.v6.2.symbols.gmt). Based on the normalized enrichment scores (NESs), the most significantly enriched signal transduction pathways were selected (Figs. 5A–5C; Tables 4 and 5). Figures 5A–5C shows that the cell cycle pathway, MCM pathway, ATR and BRCA pathway, ATM pathway, biocarta G1 pathway, E2F targets, hallmark G2/M checkpoint, and spermatogenesis, etc., were differentially enriched in phenotypes with high CELSR3 expression.

**Table 3:** Univariate and multivariate analyses of overall survival in patients with liver cancer.

**Varible**	**Univariate analysis**			**Multivariate analysis**		
	HR	*p* value	95% CI	HR	*p* value	95% CI
CELSR3 expression (high/low)	1.85	0.001^*^	1.296–2.629	1.88	0.004^*^	1.227–2.883
Age, years (≥60/<60)	1.19	0.339	0.836–1.683			
Gender (male/female)	0.80	0.218	0.562–1.141			
Family history of cancer (yes/no)	1.19	0.349	0.826–1.721			
Tumor status (with tumor/tumor free)	1.53	0.021^*^	1.068–2.200	1.14	0.544	0.750–1.724
Radiation therapy (no/yes)	0.91	0.869	0.288–2.861			
Histological type (hepatocholangio carcinoma/hepatocellular/fibrolamellar)	0.45	0.37	0.077–2.605			
Histologic grade ((G4/G3/G2/G1))	1.11	0.368	0.881–1.408			
Vascular invasion (Macro/Micro/None)	1.36	0.063	0.984–1.888			
Stage ((IV/III/II/I))	1.67	0.000^*^	1.361–2.048	1.06	0.919	0.373–2.986
T classification (TX/T4/T3/T2/T1)	1.64	0.000^*^	1.380–1.939	1.43	0.481	0.525–3.918
N classification (NX/N1/N0)	1.21	0.040^*^	1.009–1.459	1.01	0.933	0.763–1.342
M classification (MX0/M1/M0)	1.26	0.013^*^	1.050–1.518	1.17	0.297	0.869–1.585
Residual tumor (RX/R2/R1/R0)	1.48	0.001^*^	1.1791.866	1.36	0.033^*^	1.026–1.799

**Notes.**

* *P* < 0.05.

HR hazard ratio CI Confdence interval

### Validation using meta-GEO HCC cohort from GEO database and cell line data from CCLE

A total of 99 HCC specimens were obtained after combining the GSE60502 and GSE62232 microarray and batch normalization treatment. We performed enrichment analysis of meta-GEO HCC cohort data and cell line data. The results showed that after choosing the c2.cp.biocarta.v6.2.symbols.gmt gene set, two common enriched pathways,

biocarta G1 pathway, ATR and BRCA pathway, were present in both the meta-GEO cohort and the TCGA cohort of the high CELSR3 mRNA expression phenotype (Fig. 5D; Table 4). After choosing the h.all.v6.2.symbols.gmt gene set, there were two common enriched pathways, hallmark G2/M checkpoint and hallmark spermatogenesis, in the meta-GEO cohort and the TCGA cohort of the high CELSR3 mRNA expression phenotype (Fig. 5E; Table 4), and one common enriched pathway, hallmark DNA repair, in the meta-GEO cohort and the HCC cell line cohort of the high CELSR3 mRNA expression phenotype (Fig. 5G; Table 4). After choosing the c5.all.v6.2.symbols.gmt gene set, four pathways, GO somatic diversification of immune receptors, GO endonuclease activity, GO DNA repair complex and GO somatic cell DNA recombination, were differentially enriched in the meta-GEO cohort, the HCC cell line cohort and the TCGA cohort of the high CELSR3 mRNA expression phenotype (Figs. 5C, 5F, 5H; Table 5).

**Table 4:** Gene sets enriched in phenotype high.

**MSigDB collection**	**Gene set name**	**NES**	**NOM***p*-value	**FDR***q*-value
c2.cp.biocarta.v6.2.symbols.gmt				
TCGA HCC cohort	BIOCARTA_CELLCYCLE_PATHWAY	2.051	0.000	0.011
	BIOCARTA_MCM_PATHWAY	2.051	0.002	0.005
	BIOCARTA_G2_PATHWAY	1.887	0.008	0.033
	BIOCARTA_ATRBRCA_PATHWAY	1.879	0.008	0.026
	BIOCARTA_G1_PATHWAY	1.729	0.006	0.089
	BIOCARTA_ATM_PATHWAY	1.613	0.029	0.179
meta-GEO HCC cohort	BIOCARTA_MPR_PATHWAY	1.722	0.006	0.159
	BIOCARTA_G1_PATHWAY	1.520	0.021	0.211
	BIOCARTA_ATRBRCA_PATHWAY	1.547	0.023	0.213
CCLE	no			
h.all.v6.2.symbols.gmt				
TCGA HCC cohort	HALLMARK_E2F_TARGETS	2.228	0.000	0.000
	HALLMARK_G2M_CHECKPOINT	2.202	0.000	0.000
	HALLMARK_SPERMATOGENESIS	1.742	0.002	0.071
meta-GEO HCC cohort	HALLMARK_E2F_TARGETS	1.549	0.008	0.066
	HALLMARK_DNA_REPAIR	1.735	0.008	0.039
	HALLMARK_G2M_CHECKPOINT	1.565	0.016	0.095
	HALLMARK_SPERMATOGENESIS	1.560	0.017	0.075
CCLE	HALLMARK_DNA_REPAIR	1.456	0.045	0.226

**Notes.**

NES normalized enrichment score NOM nominal FDR false discovery rate TCGA The Cancer Genome Atlas GEO Gene Expression Omnibus CCLE Cancer Cell Line Encyclopedia

Gene sets with NOM *p*-val <0.05 and FDR *q*-val <0.25 are considered as significant.

**Table 5:** Gene sets enriched in phenotype high.

**MSigDB collection**	**Gene set name**	**NES**	**NOM***p*-value	FDR *q*-value
c5.all.v6.2.symbols.gmt				
TCGA HCC cohort	GO_SOMATIC_DIVERSIFICATION_OF_IMMUNE _RECEPTORS	1.843	0.013	0.032
	GO_ENDONUCLEASE_ACTIVITY	1.646	0.012	0.091
	GO_DNA_REPAIR_COMPLEX	1.787	0.024	0.044
	GO_SOMATIC_CELL_DNA_RECOMBINATION	1.644	0.036	0.092
meta-GEO HCC cohort	GO_SOMATIC_DIVERSIFICATION_OF_IMMUNE _RECEPTORS	1.706	0.018	0.087
	GO_ENDONUCLEASE_ACTIVITY	1.576	0.006	0.123
	GO_DNA_REPAIR_COMPLEX	1.759	0.006	0.086
	GO_SOMATIC_CELL_DNA_RECOMBINATION	1.548	0.039	0.134
CCLE	GO_SOMATIC_DIVERSIFICATION_OF_IMMUNE _RECEPTORS	1.693	0.000	0.181
	GO_ENDONUCLEASE_ACTIVITY	1.616	0.002	0.209
	GO_DNA_REPAIR_COMPLEX	1.598	0.010	0.212
	GO_SOMATIC_CELL_DNA_RECOMBINATION	1.602	0.014	0.219

## Discussion

This study confirmed the importance of CELSR3 in HCC and indicated that CELSR3 might serve as a biomarker of the prognosis of HCC. It also showed that high expression of CELSR3 in HCC was correlated with the age, tumor status, TNM staging, T staging, vital status, and relapse of HCC patients.

In recent years, studies examining CELSR3 have mainly focused on the effects of CELSR3 on the function of the nervous system (Zhou et al., 2009; Zhou et al., 2008; Zhou, Goffinet & Tissir, 2008) because proteins play an important role in the migration of neurons in the cortex and in the development of neuronal axons and dendrites. Recently, a relationship between CELSR3 expression and tumors, such as adult brain tumor (Katoh & Katoh, 2007) and ovarian cancer (Asad et al., 2014), has also been reported. Our study showed that CELSR3 mRNA was highly expressed in HCC, which is consistent with studies of other tumors. Interestingly, our study showed that CELSR3 mRNA was upregulated in stage I/II/III and downregulated in stage IV tumors, suggesting that CELSR3 mRNA might be differentially expressed at different stages. Alternately, the results might be due to the small sample size of stage IV patients (five cases), and thus, expanding the sample size might provide a more valid result. Because the expression of CELSR3 mRNA was higher in deceased than in surviving patients, the relationship between CELSR3 mRNA and survival must be further explored.

The role of CELSR3 in tumorigenesis and progression has also been studied. Erkan et al. (2010) suggested that tissue fibrosis is a component of chronic inflammation (liver and pancreas) and pancreatic cancer. Activated PSCs and hepatic stellate cells (HSCs) play critical roles in fibrogenesis. These researchers found that CELSR3 was selectively upregulated in stellate cells in pancreatic tumors and might provide a favorable treatment strategy for selectively targeting the tumor stroma. Scarlett et al. (2011) also observed the colocalized expression of CELSR3 with green fluorescent protein (GFP) in tumor-associated PSCs, suggesting that CELSR3 is a specific marker of circulation of bone marrow-derived (BMD) tumor-associated PSCs. In our study, the role of CELSR3 in tumorigenesis and proliferation might explain the relationship

CELSR3 is closely related to the prognosis of cancer. CELSR3 is hypermethylated in oral squamous cell carcinoma (OSCC) and can be used as a potential biomarker for the diagnosis, prognosis, and treatment of OSCC (Khor et al., 2014). Karpathakis et al. (2016) conducted a complete molecular identification of small intestinal neuroendocrine tumors (SINETs) and found that CELSR3 has a significant epigenetic mutation that might be a potential drug target. In this study, we found that patients with high CELSR3 mRNA expression had poor overall survival, especially those with histological grade G1/G2 and stage I/II, which might contribute to the precise treatment and precision targeting of HCC. Importantly, we found that CELSR3 mRNA was an independent prognostic factor for the overall survival of HCC patients and demonstrated its potential to become a biomarker for HCC. In addition, CELSR3 mRNA showed no independent prognostic significance for recurrence-free survival. However, in the subgroup analysis of histological grade G1/G2, stage I/II, and N1Nx, and M0 stages, the R0 stage was associated with a poor recurrence-free survival rate.

We performed double validation in tissue samples and cell lines for the enriched pathways of the CELSR3 high expression phenotype. The results showed that the CELSR3 high expression group showed gene enrichment in multiple different gene sets of the MSigDB database that were similar to those in the TCGA, GEO and CCLE databases. In the TCGA and GEO tissue samples, biocarta G1 pathway, ATR and BRCA pathway, E2F targets, hallmark G2/M checkpoint and hallmark spermatogenesis were differentially enriched in the CELSR3 high expression phenotype, while they were not differentially enriched in the CELSR3 high expression phenotype of the HCC cell line cohort. Whether this is related to different tissue specimens and cell lines deserves further study. Interestingly, hallmark DNA repair was differentially enriched in the CELSR3 high expression phenotype of the meta-GEO cohort and HCC cell line cohort. It is particularly noteworthy that GO somatic diversification of immune receptors, GO endonuclease activity, GO DNA repair complex and GO somatic cell DNA recombination were differentially enriched in all three cohorts. These four pathways may be important biological pathways for the involvement of CELSR3 mRNA in the pathogenesis of liver cancer and deserve further in-depth investigation.

To our knowledge, this is the first study to demonstrate the important role of CELSR3 mRNA in the prognosis of HCC. In future analyses, additional clinical trials will be required to validate the corresponding results to reveal the prognostic value of CELSR3 mRNA in HCC.

## Conclusion

Our study showed that the expression of CELSR3 mRNA was significantly increased in HCC and was correlated with some clinical features and a poor prognosis in patients. In addition, G1 pathway, ATRBRCA pathway, E2F targets, G2 M checkpoint and spermatogenesis may be the key pathways through which CELSR3 regulates liver cancer. GO somatic diversification of immune receptors, GO endonuclease activity, GO DNA repair complex and GO somatic cell DNA recombination were differentially enriched in both tissue specimens and the cell line cohort; these may be the pathways in which CELSR3 participates in the regulatory mechanism of HCC.Therefore, CELSR3 might be a valuable biomarker for HCC patients.

##  Supplemental Information

10.7717/peerj.7816/supp-1 Supplemental Information 1 The complete GSEA results of TCGA The data, heat map, Enrich the original picture and ES distribution plot of differentially enriched pathways.

10.7717/peerj.7816/supp-2 Supplemental Information 2 The complete GSEA results of GEO The data, heat map, Enrich the original picture and ES distribution plot of differentially enriched pathways.

10.7717/peerj.7816/supp-3 Supplemental Information 3 The complete GSEA results of CCLE The data, heat map, Enrich the original picture and ES distribution plot of differentially enriched pathways.

10.7717/peerj.7816/supp-4 Supplemental Information 4 GSE41804 dataset Cel data was converted using RMA and log2.

10.7717/peerj.7816/supp-5 Supplemental Information 5 GSE6764 dataset Cel data was converted using RMA and log2

10.7717/peerj.7816/supp-6 Supplemental Information 6 GSE45436 dataset Cel data was converted using RMA and log2

10.7717/peerj.7816/supp-7 Supplemental Information 7 A total of 99 HCC specimens were obtained after combining the GSE60502 and GSE62232 microarray and batch normalization treatment The liver cancer microarray GSE60502 and GSE62232 in the GEO database and performed batch normalization using the sva package of R.

10.7717/peerj.7816/supp-8 Supplemental Information 8 Raw_code For the CEL expression profiles, Robust Multi-array Average (RMA) normalization was performed using the affy package. The ggplot2 and pROC packages in the statistical software R (version 3.5.2) were used for graph generation. Discrete variables were expressed using a box plot to measure expression differences. We performed batch normalization using the sva package of R.The GSEA results were presented in the form of multipleGSEA using the Plyr, grid, gridExtra and ggplot2 packages of R.

## Additional Information and Declarations

### Data Availability

The following information was supplied regarding data availability: The raw measurements are available in the Supplemental Files.
