# Supplementary figures and images for: CELSR3 mRNA expression is increased in hepatocellular carcinoma and indicates poor prognosis
Source: PeerJ. 2019 Oct 7;7:e7816. doi: 10.7717/peerj.7816 (PMC6786253; doi:10.7717/peerj.7816)

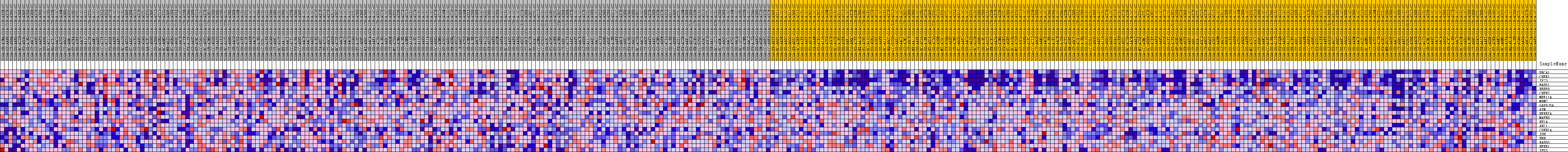

Supplement: Supplemental Information 1 — The data, heat map, Enrich the original picture and ES distribution plot of differentially enriched pathways. [file peerj-07-7816-s001.zip › TCGA/c2.cp.biocarta.v6.2.symbols.gmt/BIOCARTA_ATM_PATHWAY_141.png]

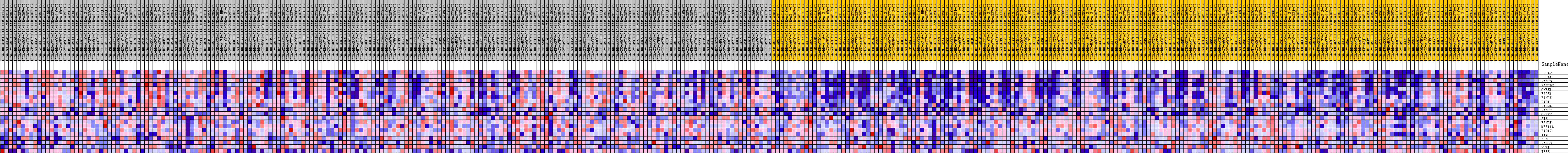

Supplement: Supplemental Information 1 — The data, heat map, Enrich the original picture and ES distribution plot of differentially enriched pathways. [file peerj-07-7816-s001.zip › TCGA/c2.cp.biocarta.v6.2.symbols.gmt/BIOCARTA_ATRBRCA_PATHWAY_135.png]

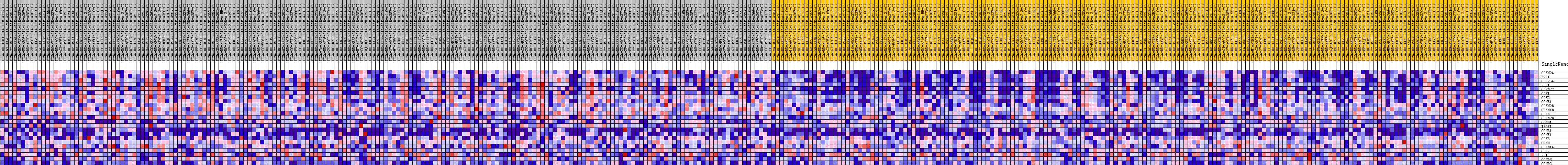

Supplement: Supplemental Information 1 — The data, heat map, Enrich the original picture and ES distribution plot of differentially enriched pathways. [file peerj-07-7816-s001.zip › TCGA/c2.cp.biocarta.v6.2.symbols.gmt/BIOCARTA_CELLCYCLE_PATHWAY_126.png]

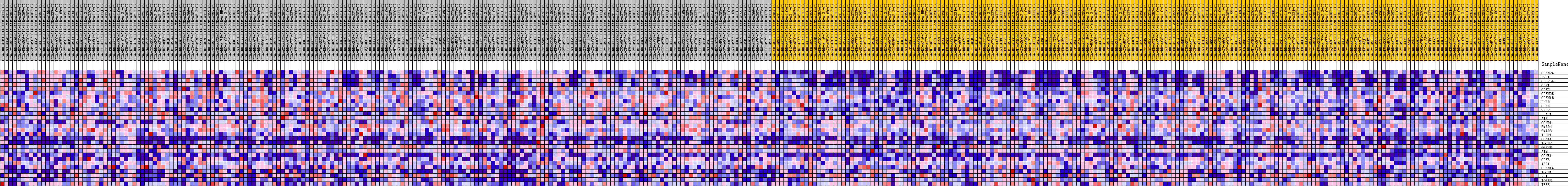

Supplement: Supplemental Information 1 — The data, heat map, Enrich the original picture and ES distribution plot of differentially enriched pathways. [file peerj-07-7816-s001.zip › TCGA/c2.cp.biocarta.v6.2.symbols.gmt/BIOCARTA_G1_PATHWAY_138.png]

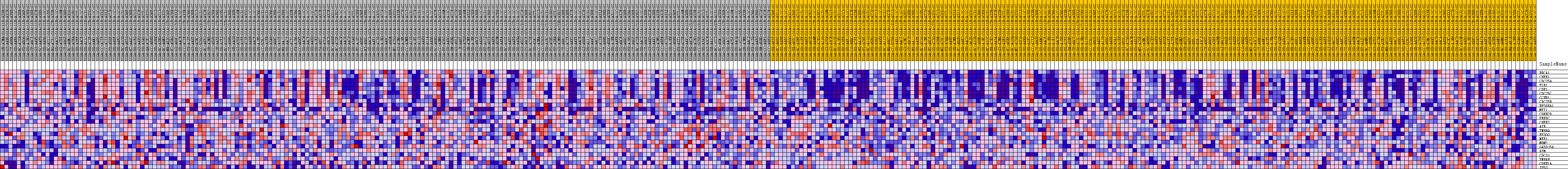

Supplement: Supplemental Information 1 — The data, heat map, Enrich the original picture and ES distribution plot of differentially enriched pathways. [file peerj-07-7816-s001.zip › TCGA/c2.cp.biocarta.v6.2.symbols.gmt/BIOCARTA_G2_PATHWAY_132.png]

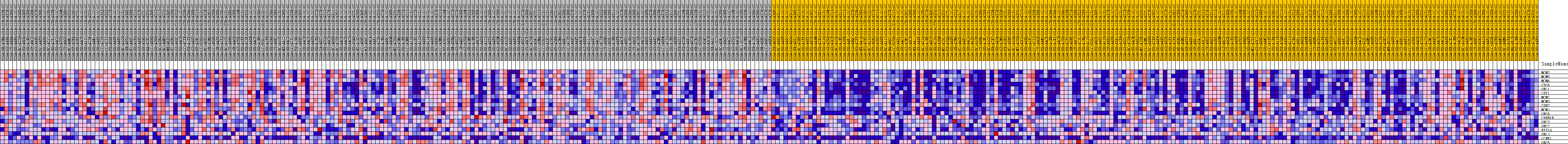

Supplement: Supplemental Information 1 — The data, heat map, Enrich the original picture and ES distribution plot of differentially enriched pathways. [file peerj-07-7816-s001.zip › TCGA/c2.cp.biocarta.v6.2.symbols.gmt/BIOCARTA_MCM_PATHWAY_129.png]

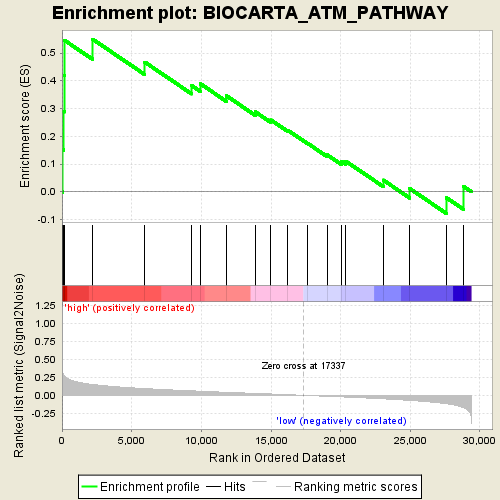

Supplement: Supplemental Information 1 — The data, heat map, Enrich the original picture and ES distribution plot of differentially enriched pathways. [file peerj-07-7816-s001.zip › TCGA/c2.cp.biocarta.v6.2.symbols.gmt/enplot_BIOCARTA_ATM_PATHWAY_140.png]

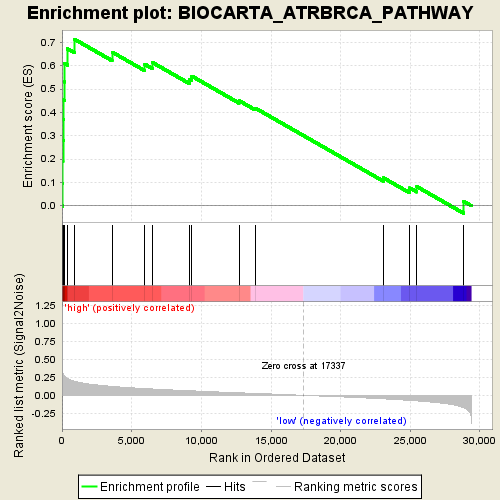

Supplement: Supplemental Information 1 — The data, heat map, Enrich the original picture and ES distribution plot of differentially enriched pathways. [file peerj-07-7816-s001.zip › TCGA/c2.cp.biocarta.v6.2.symbols.gmt/enplot_BIOCARTA_ATRBRCA_PATHWAY_134.png]

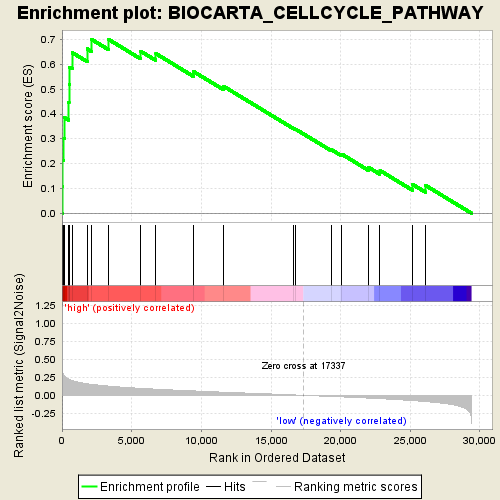

Supplement: Supplemental Information 1 — The data, heat map, Enrich the original picture and ES distribution plot of differentially enriched pathways. [file peerj-07-7816-s001.zip › TCGA/c2.cp.biocarta.v6.2.symbols.gmt/enplot_BIOCARTA_CELLCYCLE_PATHWAY_125.png]

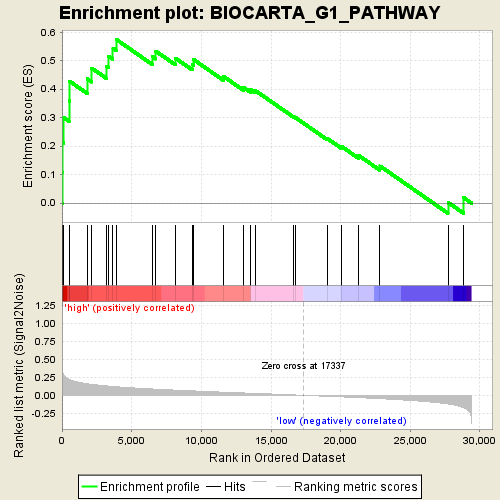

Supplement: Supplemental Information 1 — The data, heat map, Enrich the original picture and ES distribution plot of differentially enriched pathways. [file peerj-07-7816-s001.zip › TCGA/c2.cp.biocarta.v6.2.symbols.gmt/enplot_BIOCARTA_G1_PATHWAY_137.png]

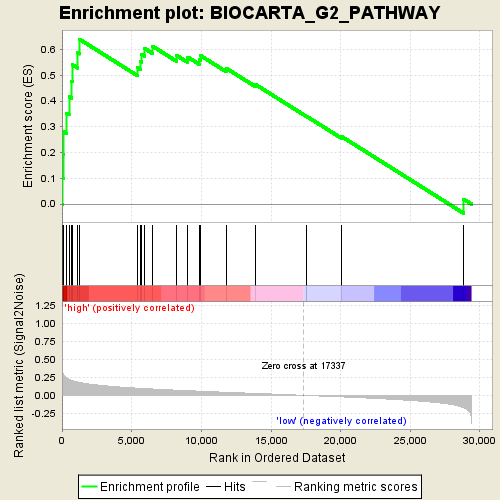

Supplement: Supplemental Information 1 — The data, heat map, Enrich the original picture and ES distribution plot of differentially enriched pathways. [file peerj-07-7816-s001.zip › TCGA/c2.cp.biocarta.v6.2.symbols.gmt/enplot_BIOCARTA_G2_PATHWAY_131.png]

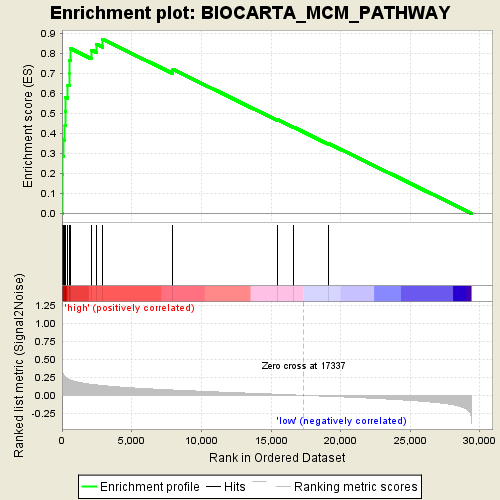

Supplement: Supplemental Information 1 — The data, heat map, Enrich the original picture and ES distribution plot of differentially enriched pathways. [file peerj-07-7816-s001.zip › TCGA/c2.cp.biocarta.v6.2.symbols.gmt/enplot_BIOCARTA_MCM_PATHWAY_128.png]

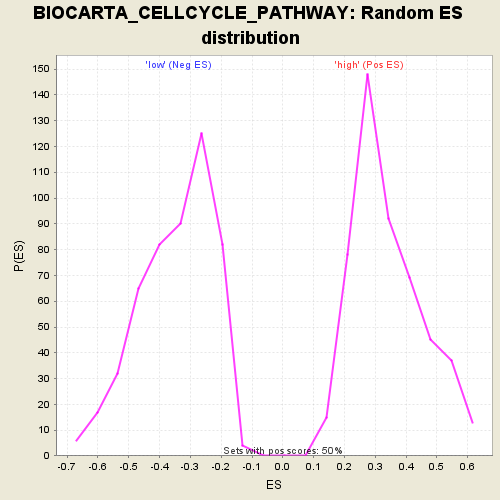

Supplement: Supplemental Information 1 — The data, heat map, Enrich the original picture and ES distribution plot of differentially enriched pathways. [file peerj-07-7816-s001.zip › TCGA/c2.cp.biocarta.v6.2.symbols.gmt/gset_rnd_es_dist_127.png]

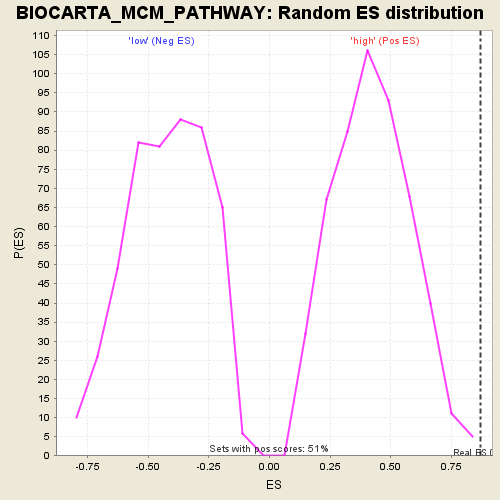

Supplement: Supplemental Information 1 — The data, heat map, Enrich the original picture and ES distribution plot of differentially enriched pathways. [file peerj-07-7816-s001.zip › TCGA/c2.cp.biocarta.v6.2.symbols.gmt/gset_rnd_es_dist_130.png]

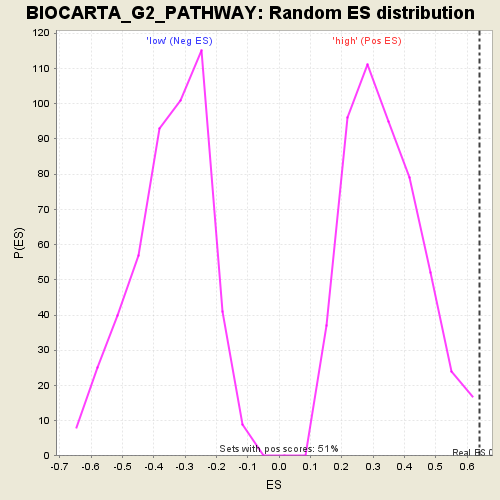

Supplement: Supplemental Information 1 — The data, heat map, Enrich the original picture and ES distribution plot of differentially enriched pathways. [file peerj-07-7816-s001.zip › TCGA/c2.cp.biocarta.v6.2.symbols.gmt/gset_rnd_es_dist_133.png]

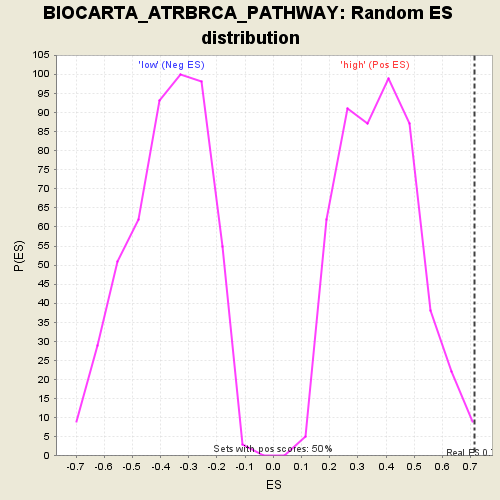

Supplement: Supplemental Information 1 — The data, heat map, Enrich the original picture and ES distribution plot of differentially enriched pathways. [file peerj-07-7816-s001.zip › TCGA/c2.cp.biocarta.v6.2.symbols.gmt/gset_rnd_es_dist_136.png]

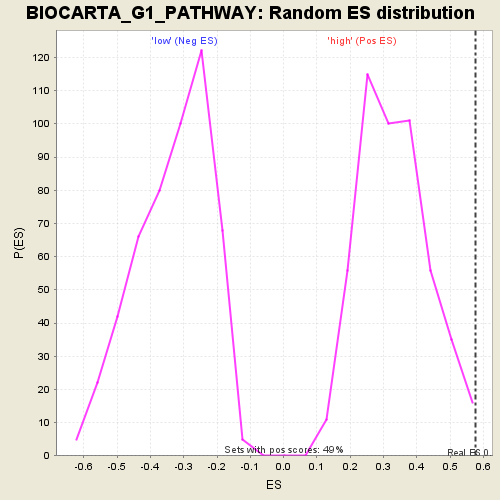

Supplement: Supplemental Information 1 — The data, heat map, Enrich the original picture and ES distribution plot of differentially enriched pathways. [file peerj-07-7816-s001.zip › TCGA/c2.cp.biocarta.v6.2.symbols.gmt/gset_rnd_es_dist_139.png]

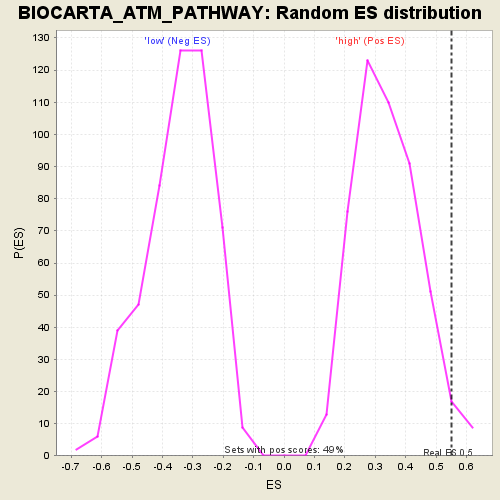

Supplement: Supplemental Information 1 — The data, heat map, Enrich the original picture and ES distribution plot of differentially enriched pathways. [file peerj-07-7816-s001.zip › TCGA/c2.cp.biocarta.v6.2.symbols.gmt/gset_rnd_es_dist_142.png]

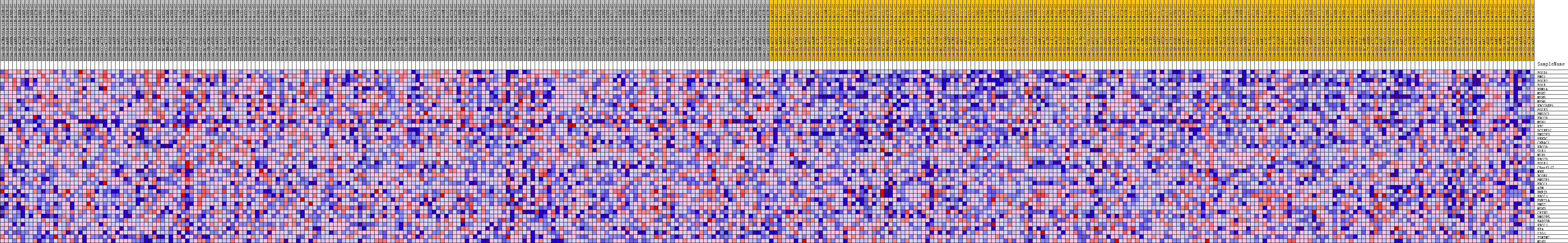

Supplement: Supplemental Information 1 — The data, heat map, Enrich the original picture and ES distribution plot of differentially enriched pathways. [file peerj-07-7816-s001.zip › TCGA/c5.all.v6.2.symbols.gmt/GO_DNA_REPAIR_COMPLEX_547.png]

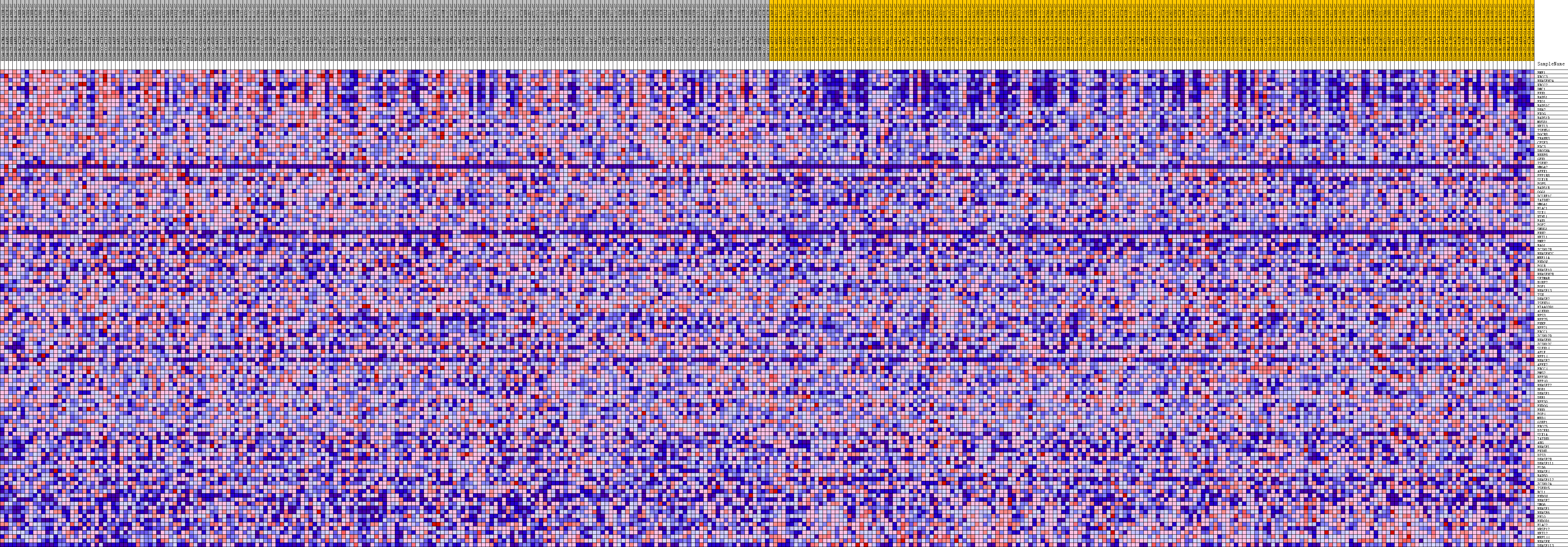

Supplement: Supplemental Information 1 — The data, heat map, Enrich the original picture and ES distribution plot of differentially enriched pathways. [file peerj-07-7816-s001.zip › TCGA/c5.all.v6.2.symbols.gmt/GO_ENDONUCLEASE_ACTIVITY_721.png]

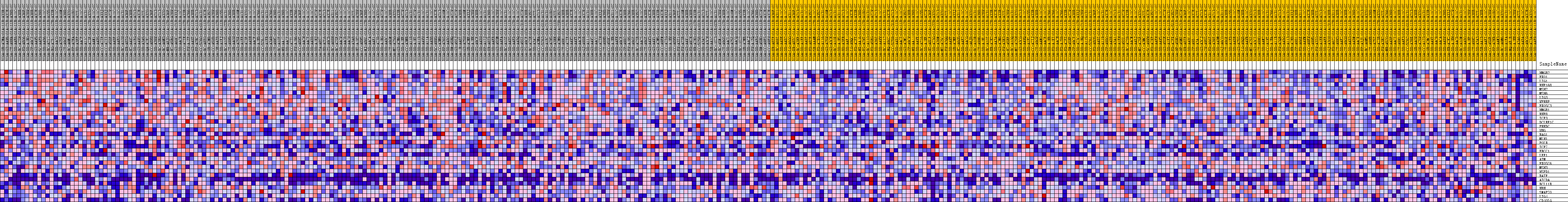

Supplement: Supplemental Information 1 — The data, heat map, Enrich the original picture and ES distribution plot of differentially enriched pathways. [file peerj-07-7816-s001.zip › TCGA/c5.all.v6.2.symbols.gmt/GO_SOMATIC_CELL_DNA_RECOMBINATION_697.png]

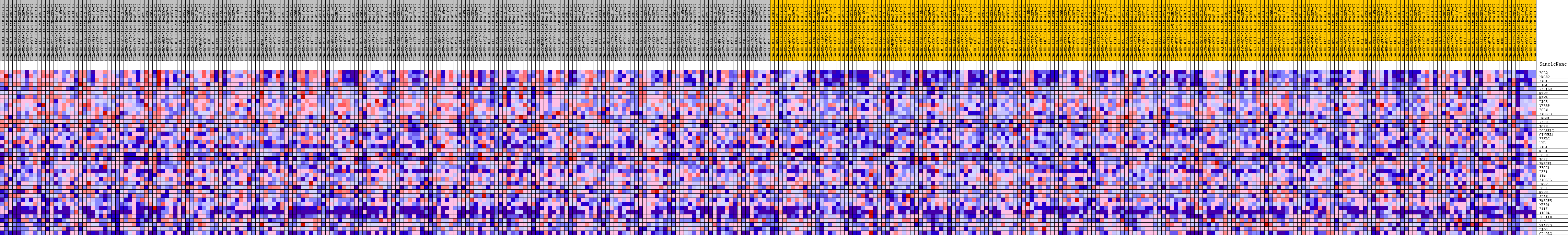

Supplement: Supplemental Information 1 — The data, heat map, Enrich the original picture and ES distribution plot of differentially enriched pathways. [file peerj-07-7816-s001.zip › TCGA/c5.all.v6.2.symbols.gmt/GO_SOMATIC_DIVERSIFICATION_OF_IMMUNE_RECEPTORS_343.png]

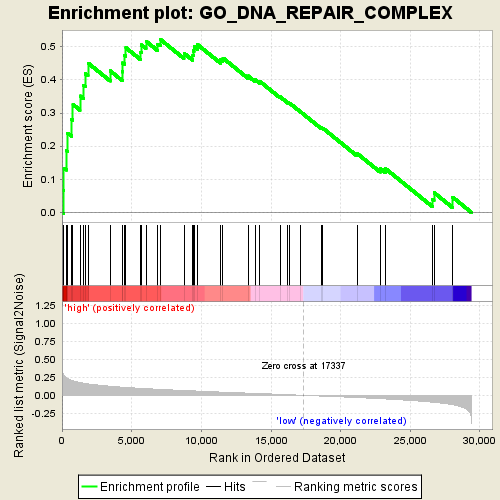

Supplement: Supplemental Information 1 — The data, heat map, Enrich the original picture and ES distribution plot of differentially enriched pathways. [file peerj-07-7816-s001.zip › TCGA/c5.all.v6.2.symbols.gmt/enplot_GO_DNA_REPAIR_COMPLEX_546.png]

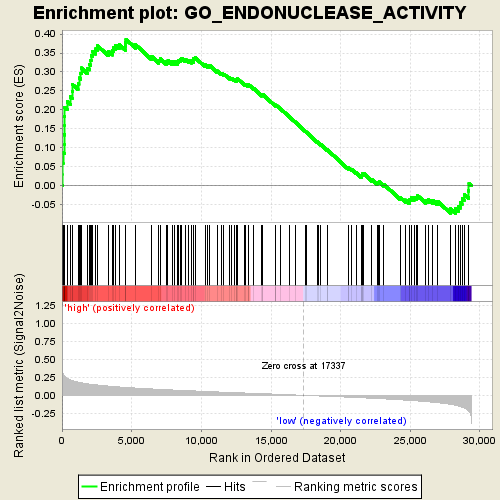

Supplement: Supplemental Information 1 — The data, heat map, Enrich the original picture and ES distribution plot of differentially enriched pathways. [file peerj-07-7816-s001.zip › TCGA/c5.all.v6.2.symbols.gmt/enplot_GO_ENDONUCLEASE_ACTIVITY_720.png]

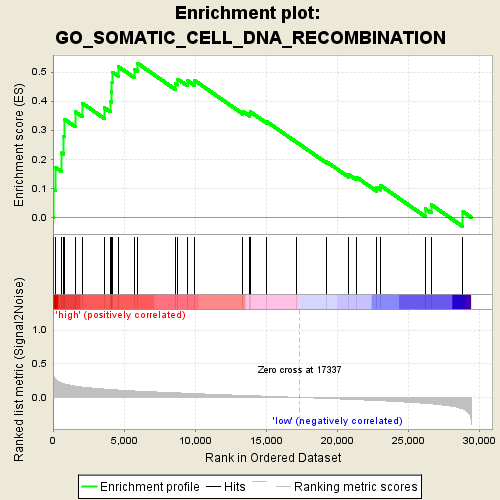

Supplement: Supplemental Information 1 — The data, heat map, Enrich the original picture and ES distribution plot of differentially enriched pathways. [file peerj-07-7816-s001.zip › TCGA/c5.all.v6.2.symbols.gmt/enplot_GO_SOMATIC_CELL_DNA_RECOMBINATION_696.png]

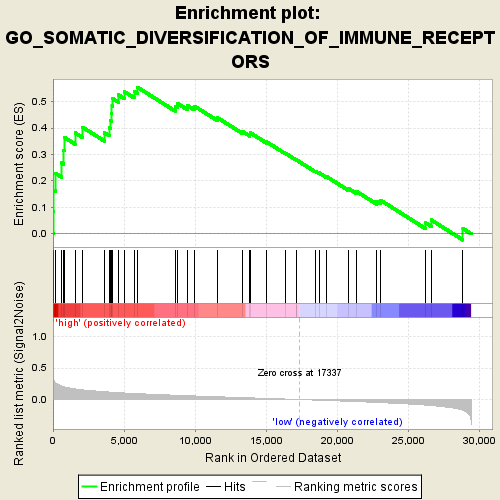

Supplement: Supplemental Information 1 — The data, heat map, Enrich the original picture and ES distribution plot of differentially enriched pathways. [file peerj-07-7816-s001.zip › TCGA/c5.all.v6.2.symbols.gmt/enplot_GO_SOMATIC_DIVERSIFICATION_OF_IMMUNE_RECEPTORS_342.png]

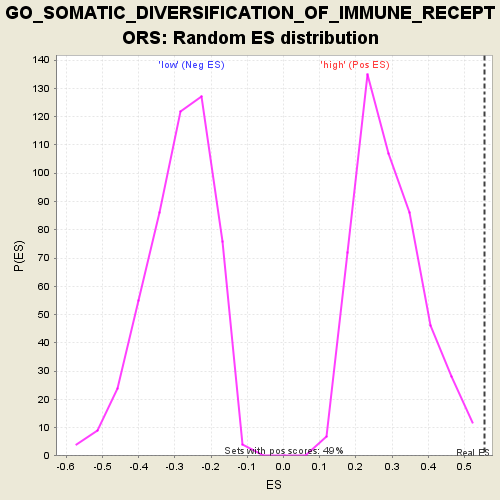

Supplement: Supplemental Information 1 — The data, heat map, Enrich the original picture and ES distribution plot of differentially enriched pathways. [file peerj-07-7816-s001.zip › TCGA/c5.all.v6.2.symbols.gmt/gset_rnd_es_dist_344.png]

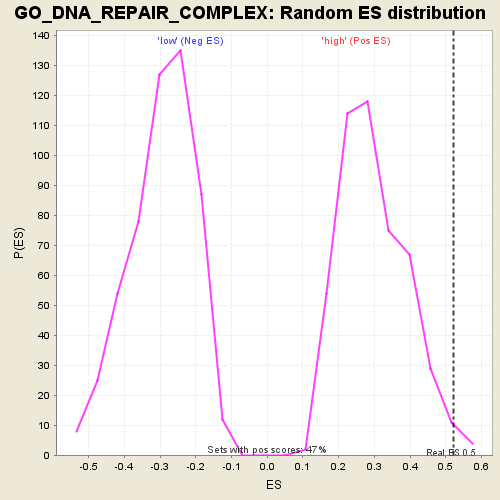

Supplement: Supplemental Information 1 — The data, heat map, Enrich the original picture and ES distribution plot of differentially enriched pathways. [file peerj-07-7816-s001.zip › TCGA/c5.all.v6.2.symbols.gmt/gset_rnd_es_dist_548.png]

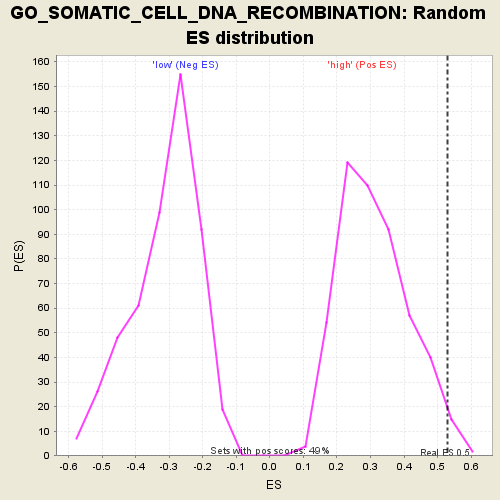

Supplement: Supplemental Information 1 — The data, heat map, Enrich the original picture and ES distribution plot of differentially enriched pathways. [file peerj-07-7816-s001.zip › TCGA/c5.all.v6.2.symbols.gmt/gset_rnd_es_dist_698.png]

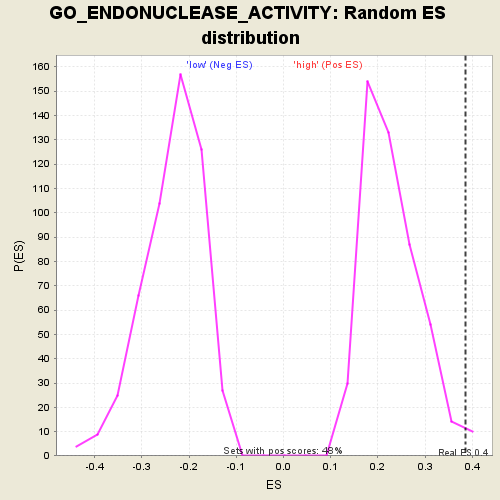

Supplement: Supplemental Information 1 — The data, heat map, Enrich the original picture and ES distribution plot of differentially enriched pathways. [file peerj-07-7816-s001.zip › TCGA/c5.all.v6.2.symbols.gmt/gset_rnd_es_dist_722.png]

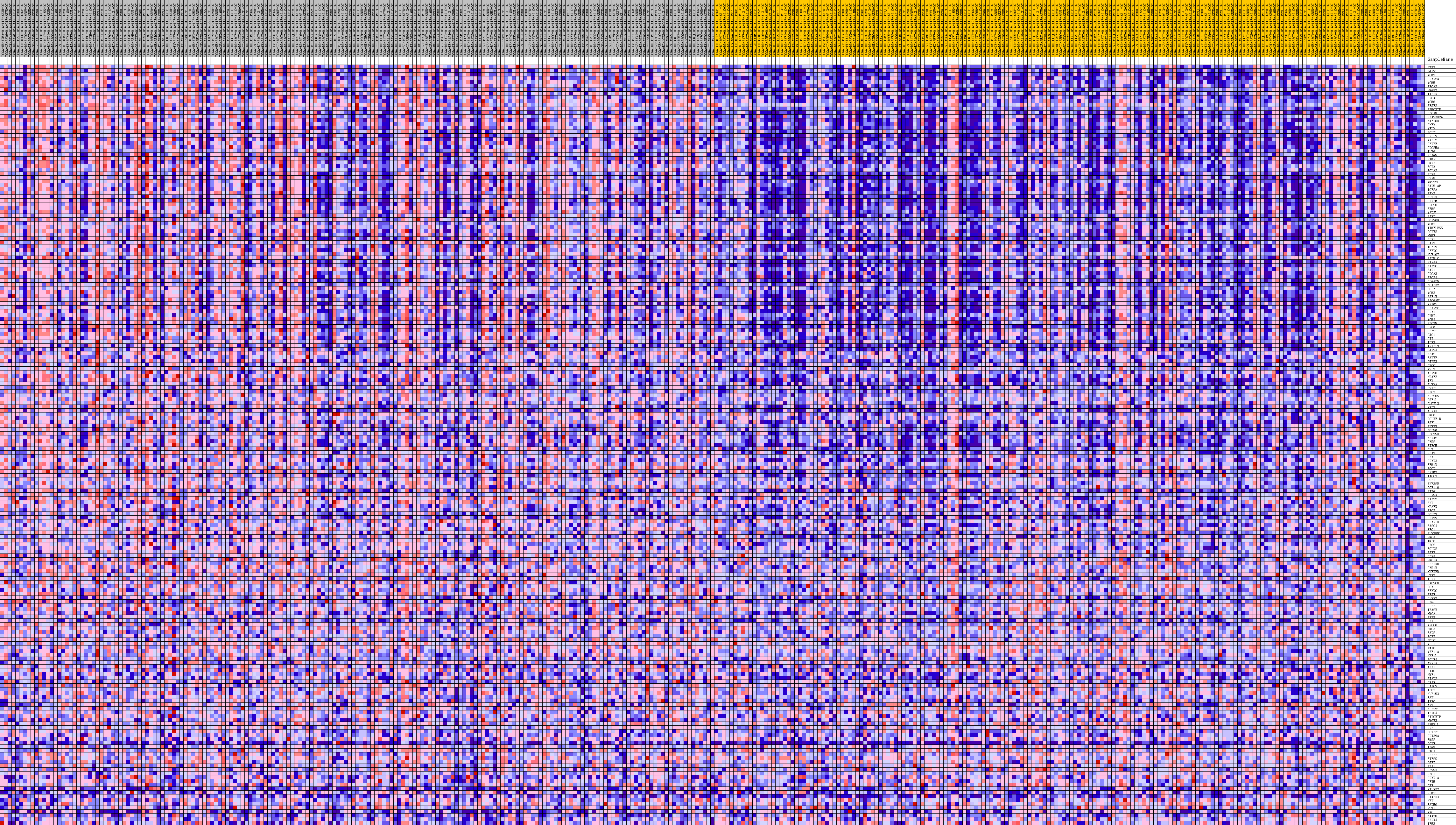

Supplement: Supplemental Information 1 — The data, heat map, Enrich the original picture and ES distribution plot of differentially enriched pathways. [file peerj-07-7816-s001.zip › TCGA/h.all.v6.2.symbols.gmt/HALLMARK_E2F_TARGETS_4.png]

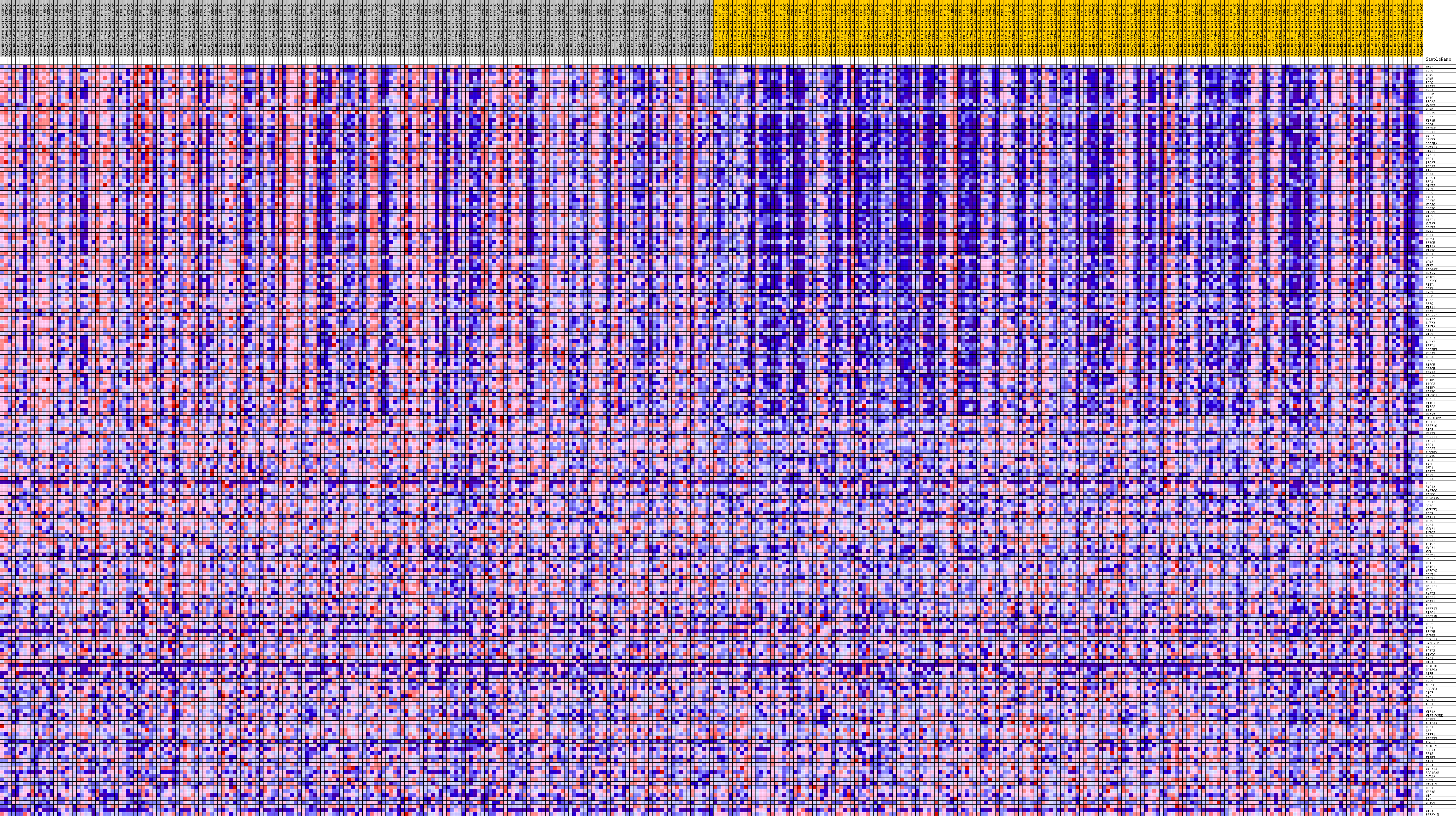

Supplement: Supplemental Information 1 — The data, heat map, Enrich the original picture and ES distribution plot of differentially enriched pathways. [file peerj-07-7816-s001.zip › TCGA/h.all.v6.2.symbols.gmt/HALLMARK_G2M_CHECKPOINT_7.png]

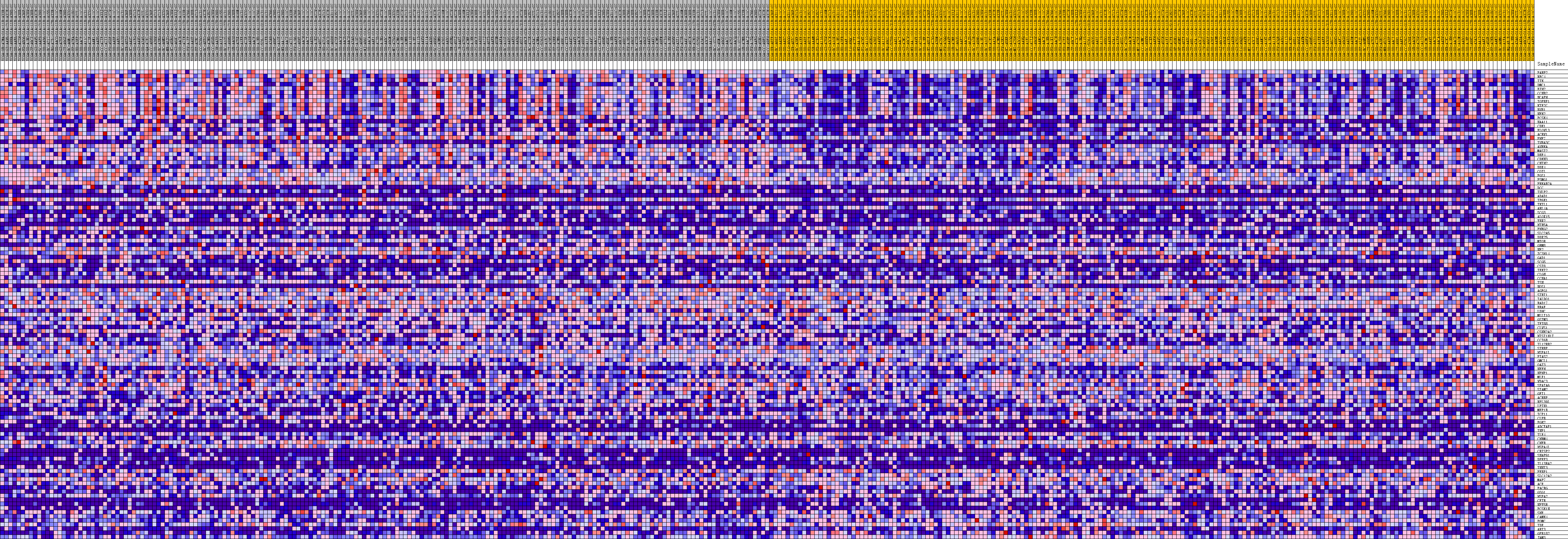

Supplement: Supplemental Information 1 — The data, heat map, Enrich the original picture and ES distribution plot of differentially enriched pathways. [file peerj-07-7816-s001.zip › TCGA/h.all.v6.2.symbols.gmt/HALLMARK_SPERMATOGENESIS_10.png]

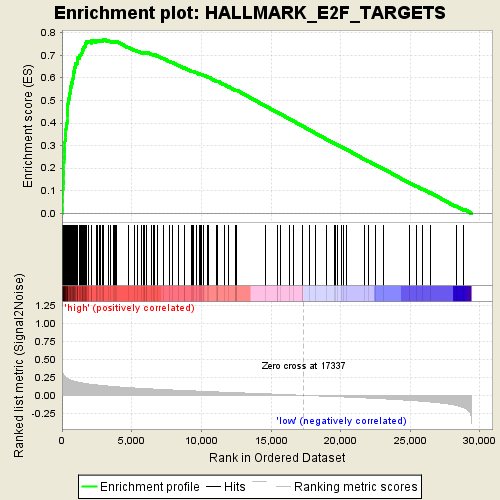

Supplement: Supplemental Information 1 — The data, heat map, Enrich the original picture and ES distribution plot of differentially enriched pathways. [file peerj-07-7816-s001.zip › TCGA/h.all.v6.2.symbols.gmt/enplot_HALLMARK_E2F_TARGETS_3.png]

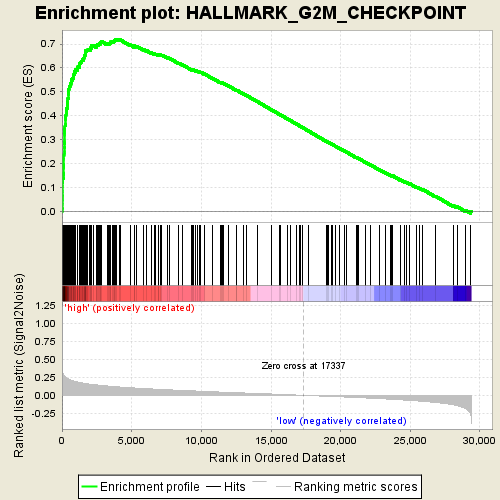

Supplement: Supplemental Information 1 — The data, heat map, Enrich the original picture and ES distribution plot of differentially enriched pathways. [file peerj-07-7816-s001.zip › TCGA/h.all.v6.2.symbols.gmt/enplot_HALLMARK_G2M_CHECKPOINT_6.png]

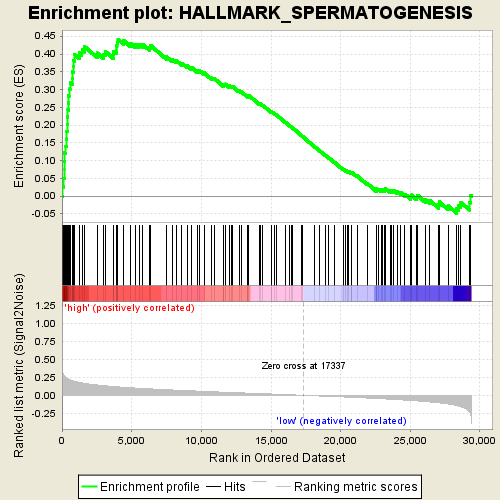

Supplement: Supplemental Information 1 — The data, heat map, Enrich the original picture and ES distribution plot of differentially enriched pathways. [file peerj-07-7816-s001.zip › TCGA/h.all.v6.2.symbols.gmt/enplot_HALLMARK_SPERMATOGENESIS_9.png]

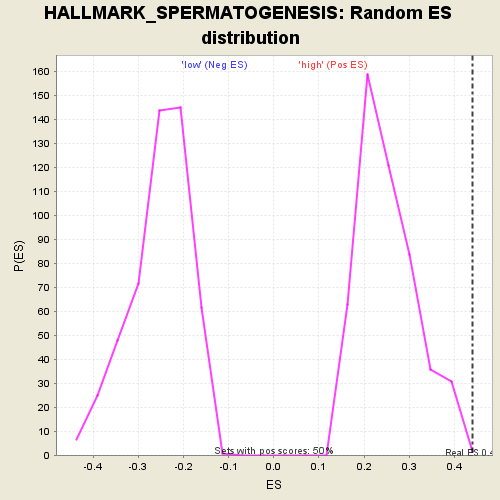

Supplement: Supplemental Information 1 — The data, heat map, Enrich the original picture and ES distribution plot of differentially enriched pathways. [file peerj-07-7816-s001.zip › TCGA/h.all.v6.2.symbols.gmt/gset_rnd_es_dist_11.png]

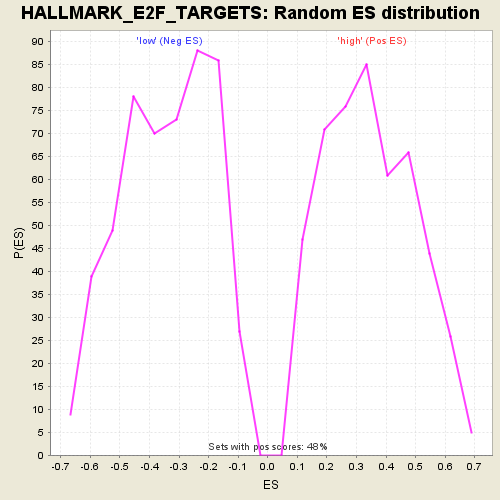

Supplement: Supplemental Information 1 — The data, heat map, Enrich the original picture and ES distribution plot of differentially enriched pathways. [file peerj-07-7816-s001.zip › TCGA/h.all.v6.2.symbols.gmt/gset_rnd_es_dist_5.png]

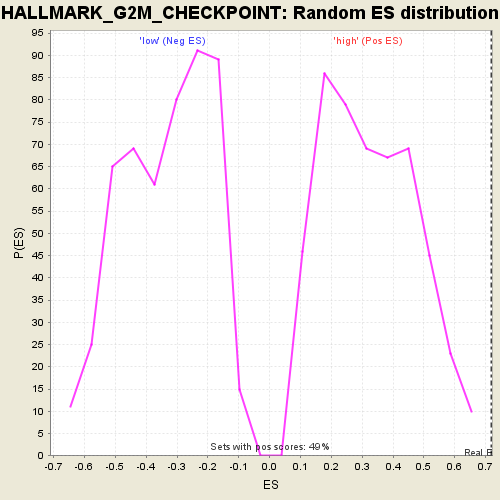

Supplement: Supplemental Information 1 — The data, heat map, Enrich the original picture and ES distribution plot of differentially enriched pathways. [file peerj-07-7816-s001.zip › TCGA/h.all.v6.2.symbols.gmt/gset_rnd_es_dist_8.png]

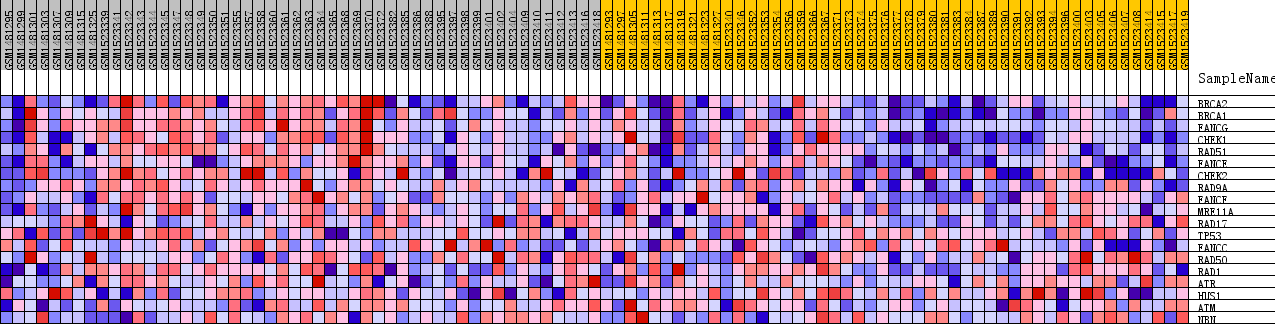

Supplement: Supplemental Information 2 — The data, heat map, Enrich the original picture and ES distribution plot of differentially enriched pathways. [file peerj-07-7816-s002.zip › GEO/c2.cp.biocarta.v6.2.symbols.gmt/BIOCARTA_ATRBRCA_PATHWAY_165.png]

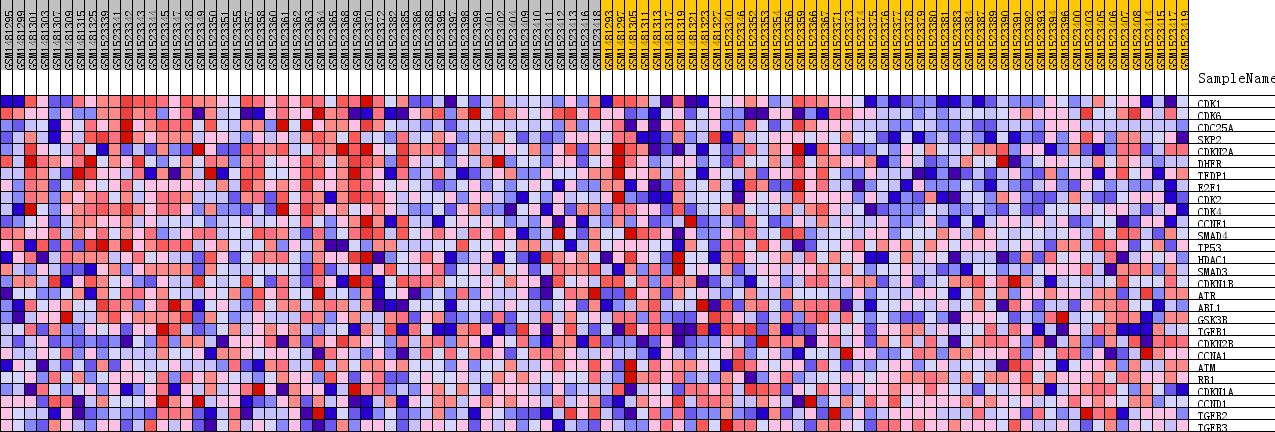

Supplement: Supplemental Information 2 — The data, heat map, Enrich the original picture and ES distribution plot of differentially enriched pathways. [file peerj-07-7816-s002.zip › GEO/c2.cp.biocarta.v6.2.symbols.gmt/BIOCARTA_G1_PATHWAY_168.png]

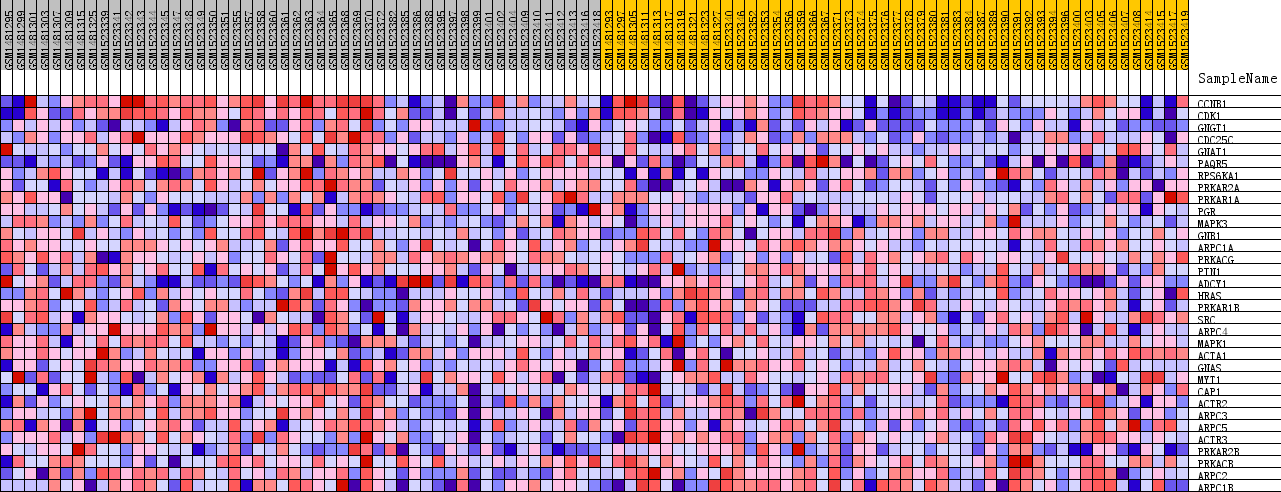

Supplement: Supplemental Information 2 — The data, heat map, Enrich the original picture and ES distribution plot of differentially enriched pathways. [file peerj-07-7816-s002.zip › GEO/c2.cp.biocarta.v6.2.symbols.gmt/BIOCARTA_MPR_PATHWAY_156.png]

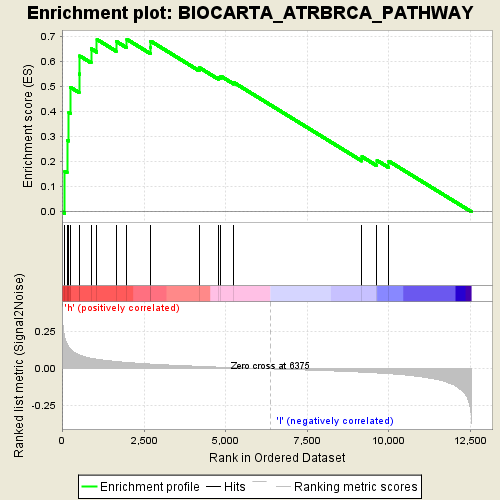

Supplement: Supplemental Information 2 — The data, heat map, Enrich the original picture and ES distribution plot of differentially enriched pathways. [file peerj-07-7816-s002.zip › GEO/c2.cp.biocarta.v6.2.symbols.gmt/enplot_BIOCARTA_ATRBRCA_PATHWAY_164.png]

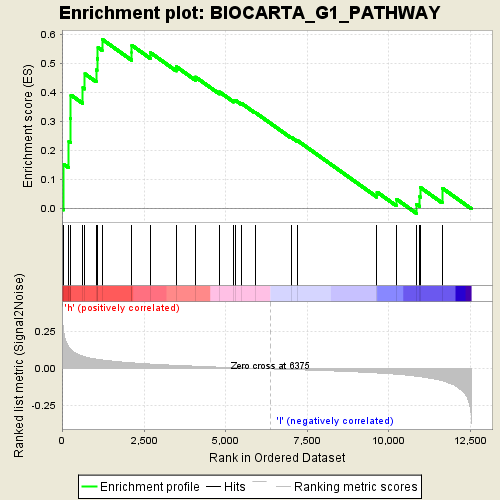

Supplement: Supplemental Information 2 — The data, heat map, Enrich the original picture and ES distribution plot of differentially enriched pathways. [file peerj-07-7816-s002.zip › GEO/c2.cp.biocarta.v6.2.symbols.gmt/enplot_BIOCARTA_G1_PATHWAY_167.png]

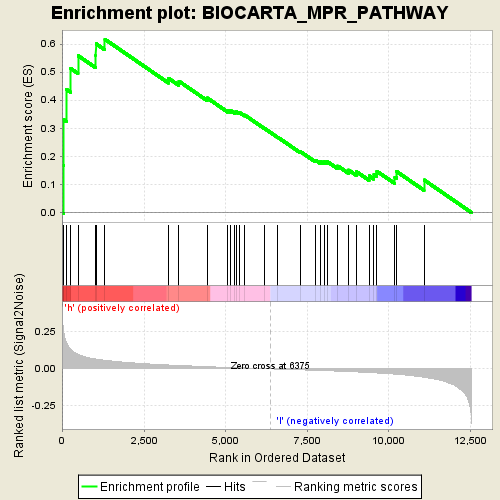

Supplement: Supplemental Information 2 — The data, heat map, Enrich the original picture and ES distribution plot of differentially enriched pathways. [file peerj-07-7816-s002.zip › GEO/c2.cp.biocarta.v6.2.symbols.gmt/enplot_BIOCARTA_MPR_PATHWAY_155.png]

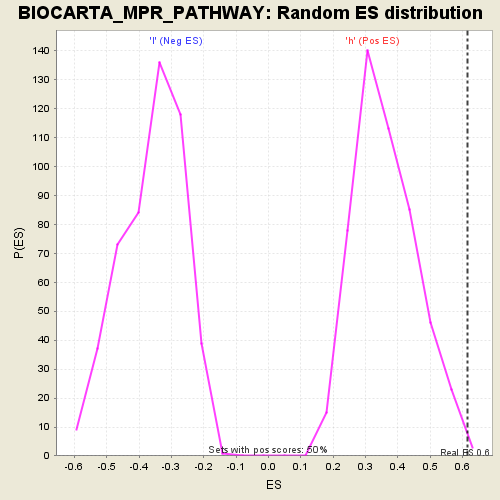

Supplement: Supplemental Information 2 — The data, heat map, Enrich the original picture and ES distribution plot of differentially enriched pathways. [file peerj-07-7816-s002.zip › GEO/c2.cp.biocarta.v6.2.symbols.gmt/gset_rnd_es_dist_157.png]

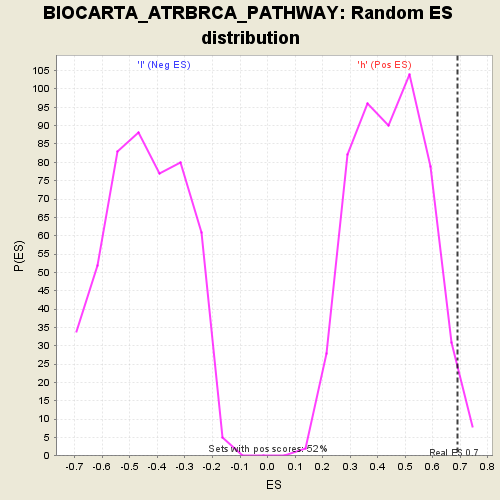

Supplement: Supplemental Information 2 — The data, heat map, Enrich the original picture and ES distribution plot of differentially enriched pathways. [file peerj-07-7816-s002.zip › GEO/c2.cp.biocarta.v6.2.symbols.gmt/gset_rnd_es_dist_166.png]

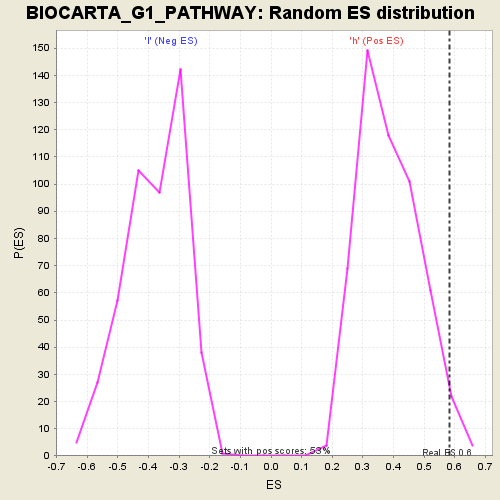

Supplement: Supplemental Information 2 — The data, heat map, Enrich the original picture and ES distribution plot of differentially enriched pathways. [file peerj-07-7816-s002.zip › GEO/c2.cp.biocarta.v6.2.symbols.gmt/gset_rnd_es_dist_169.png]

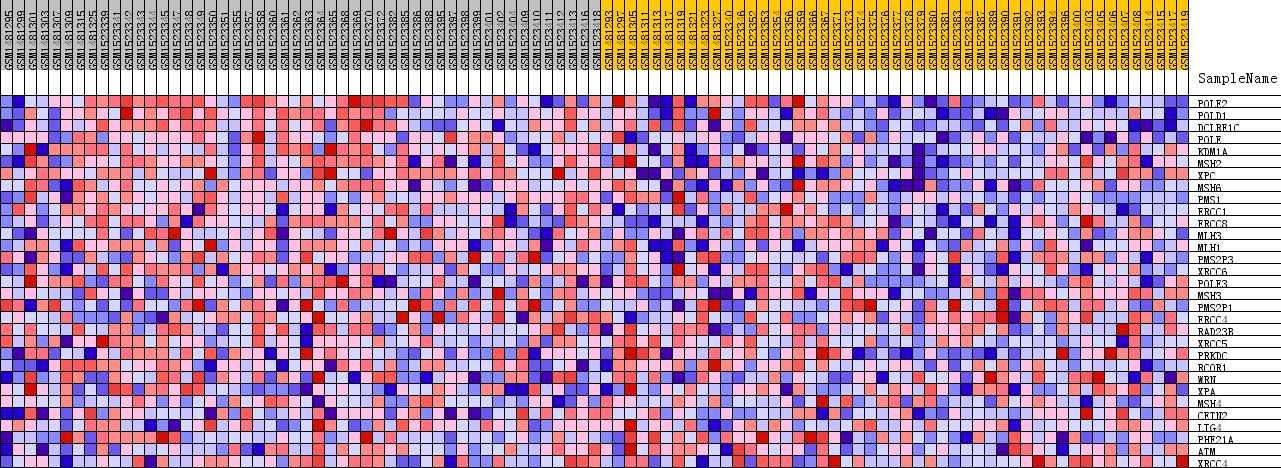

Supplement: Supplemental Information 2 — The data, heat map, Enrich the original picture and ES distribution plot of differentially enriched pathways. [file peerj-07-7816-s002.zip › GEO/c5.all.v6.2.symbols.gmt/GO_DNA_REPAIR_COMPLEX_169.png]

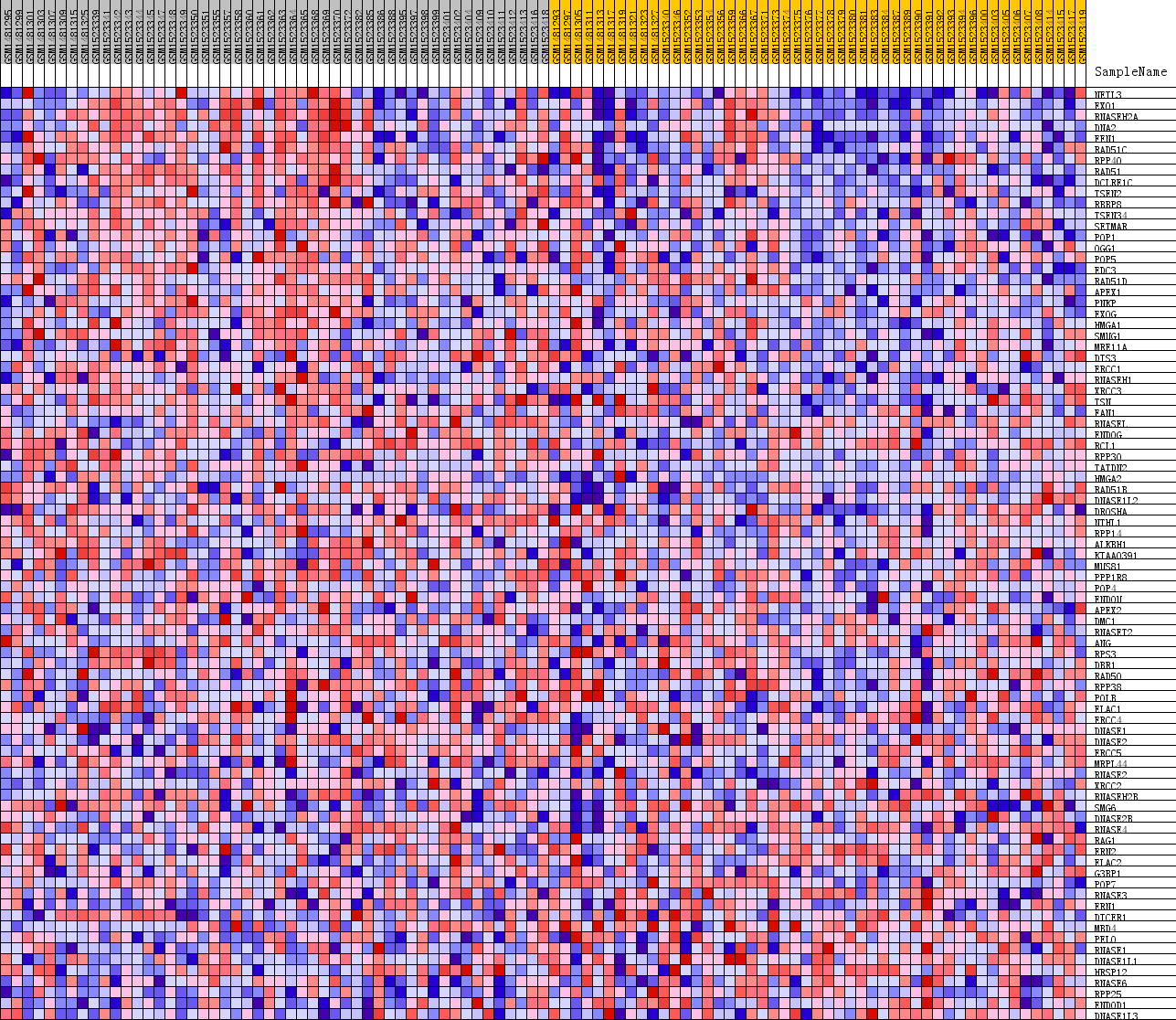

Supplement: Supplemental Information 2 — The data, heat map, Enrich the original picture and ES distribution plot of differentially enriched pathways. [file peerj-07-7816-s002.zip › GEO/c5.all.v6.2.symbols.gmt/GO_ENDONUCLEASE_ACTIVITY_703.png]

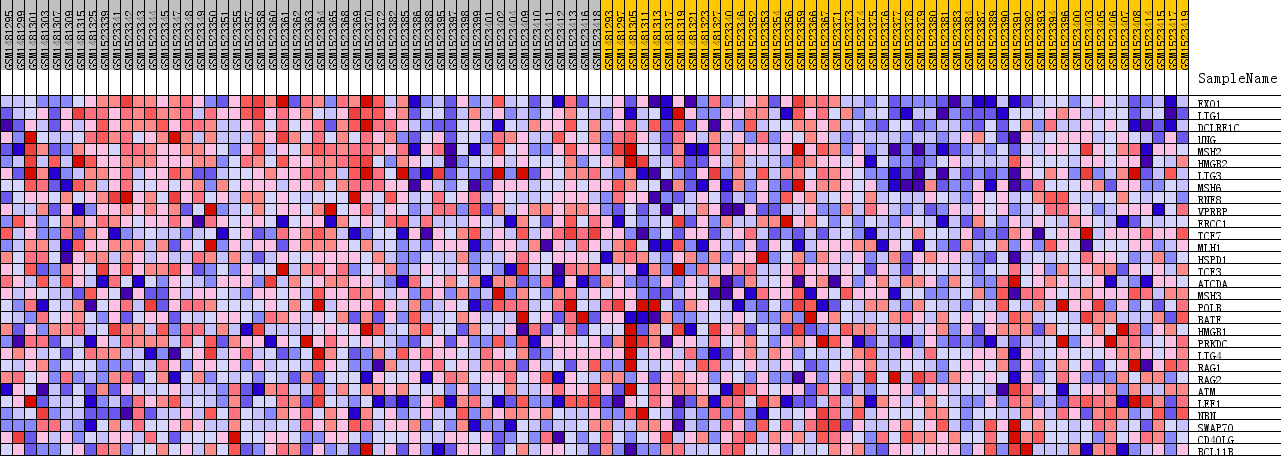

Supplement: Supplemental Information 2 — The data, heat map, Enrich the original picture and ES distribution plot of differentially enriched pathways. [file peerj-07-7816-s002.zip › GEO/c5.all.v6.2.symbols.gmt/GO_SOMATIC_CELL_DNA_RECOMBINATION_829.png]

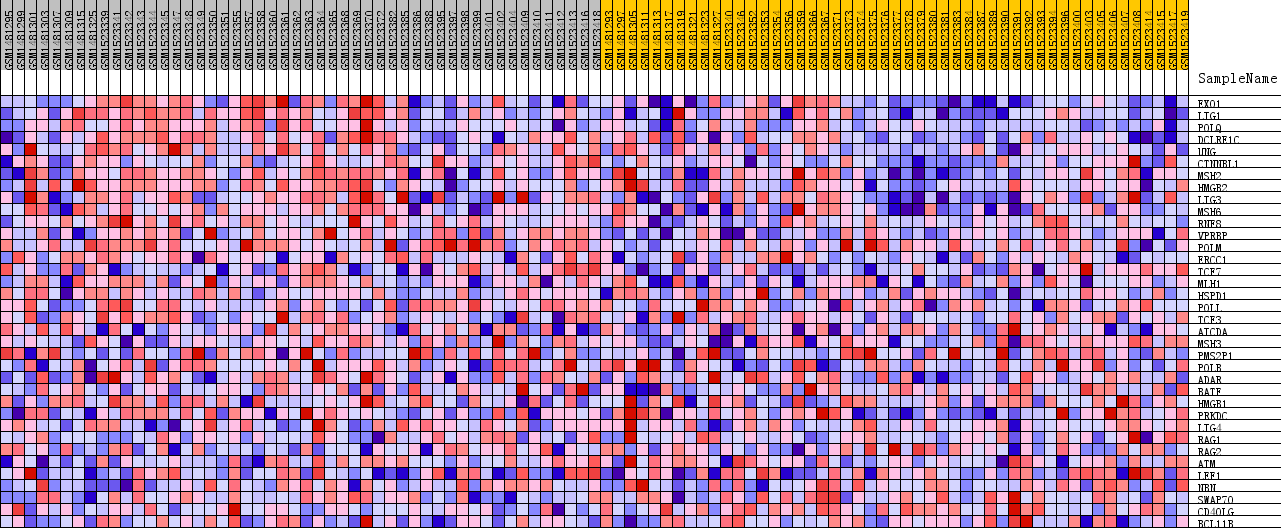

Supplement: Supplemental Information 2 — The data, heat map, Enrich the original picture and ES distribution plot of differentially enriched pathways. [file peerj-07-7816-s002.zip › GEO/c5.all.v6.2.symbols.gmt/GO_SOMATIC_DIVERSIFICATION_OF_IMMUNE_RECEPTORS_304.png]

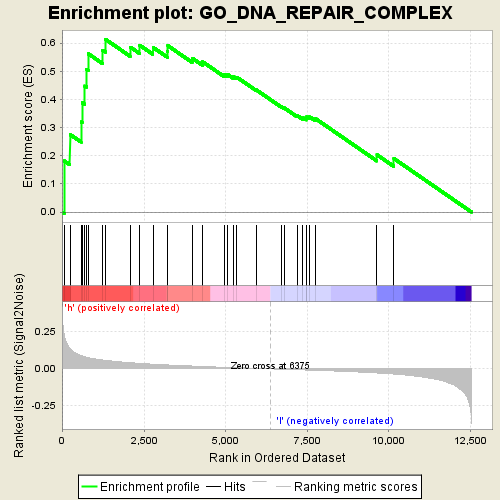

Supplement: Supplemental Information 2 — The data, heat map, Enrich the original picture and ES distribution plot of differentially enriched pathways. [file peerj-07-7816-s002.zip › GEO/c5.all.v6.2.symbols.gmt/enplot_GO_DNA_REPAIR_COMPLEX_168.png]

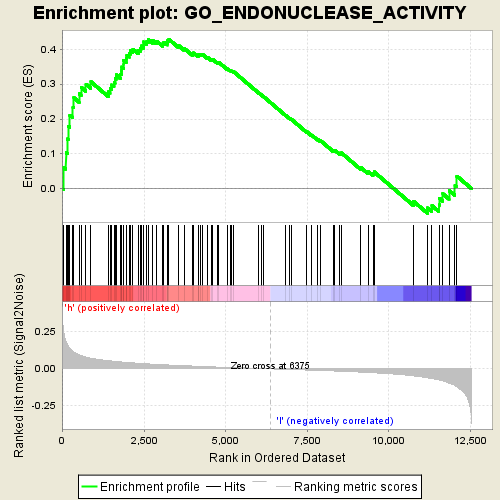

Supplement: Supplemental Information 2 — The data, heat map, Enrich the original picture and ES distribution plot of differentially enriched pathways. [file peerj-07-7816-s002.zip › GEO/c5.all.v6.2.symbols.gmt/enplot_GO_ENDONUCLEASE_ACTIVITY_702.png]

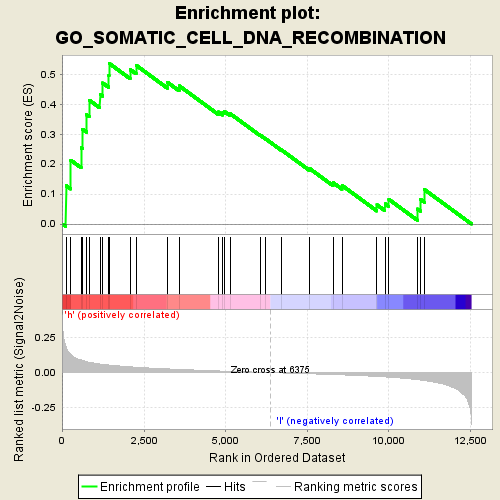

Supplement: Supplemental Information 2 — The data, heat map, Enrich the original picture and ES distribution plot of differentially enriched pathways. [file peerj-07-7816-s002.zip › GEO/c5.all.v6.2.symbols.gmt/enplot_GO_SOMATIC_CELL_DNA_RECOMBINATION_828.png]

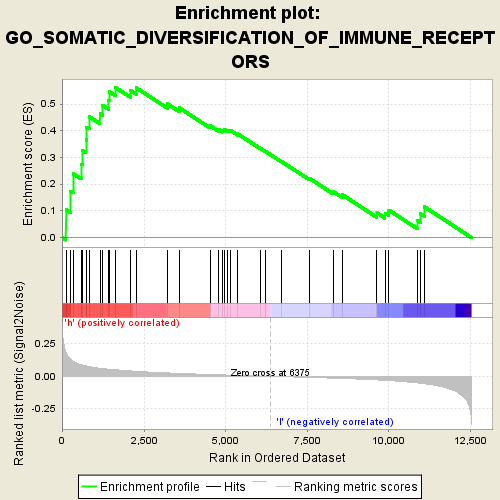

Supplement: Supplemental Information 2 — The data, heat map, Enrich the original picture and ES distribution plot of differentially enriched pathways. [file peerj-07-7816-s002.zip › GEO/c5.all.v6.2.symbols.gmt/enplot_GO_SOMATIC_DIVERSIFICATION_OF_IMMUNE_RECEPTORS_303.png]

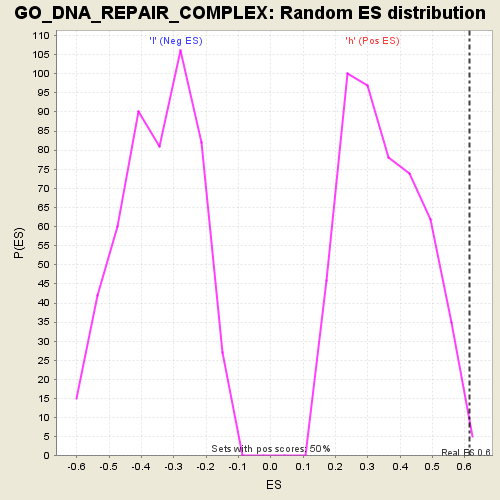

Supplement: Supplemental Information 2 — The data, heat map, Enrich the original picture and ES distribution plot of differentially enriched pathways. [file peerj-07-7816-s002.zip › GEO/c5.all.v6.2.symbols.gmt/gset_rnd_es_dist_170.png]

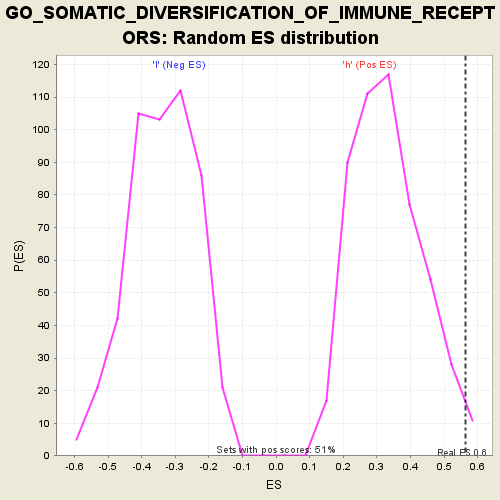

Supplement: Supplemental Information 2 — The data, heat map, Enrich the original picture and ES distribution plot of differentially enriched pathways. [file peerj-07-7816-s002.zip › GEO/c5.all.v6.2.symbols.gmt/gset_rnd_es_dist_305.png]

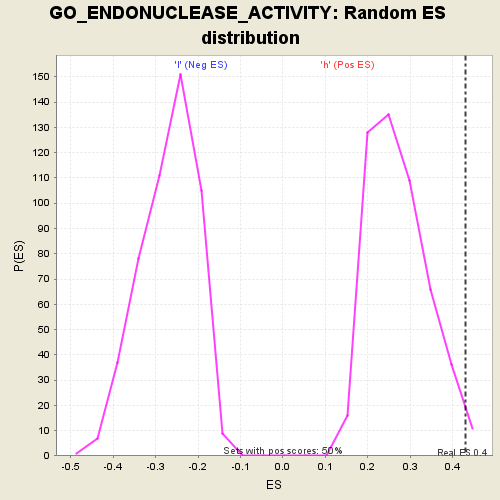

Supplement: Supplemental Information 2 — The data, heat map, Enrich the original picture and ES distribution plot of differentially enriched pathways. [file peerj-07-7816-s002.zip › GEO/c5.all.v6.2.symbols.gmt/gset_rnd_es_dist_704.png]

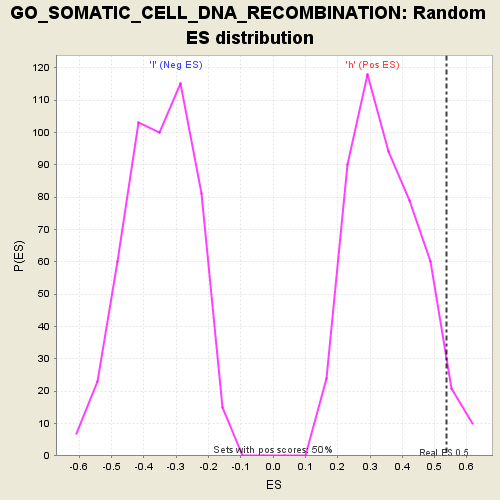

Supplement: Supplemental Information 2 — The data, heat map, Enrich the original picture and ES distribution plot of differentially enriched pathways. [file peerj-07-7816-s002.zip › GEO/c5.all.v6.2.symbols.gmt/gset_rnd_es_dist_830.png]

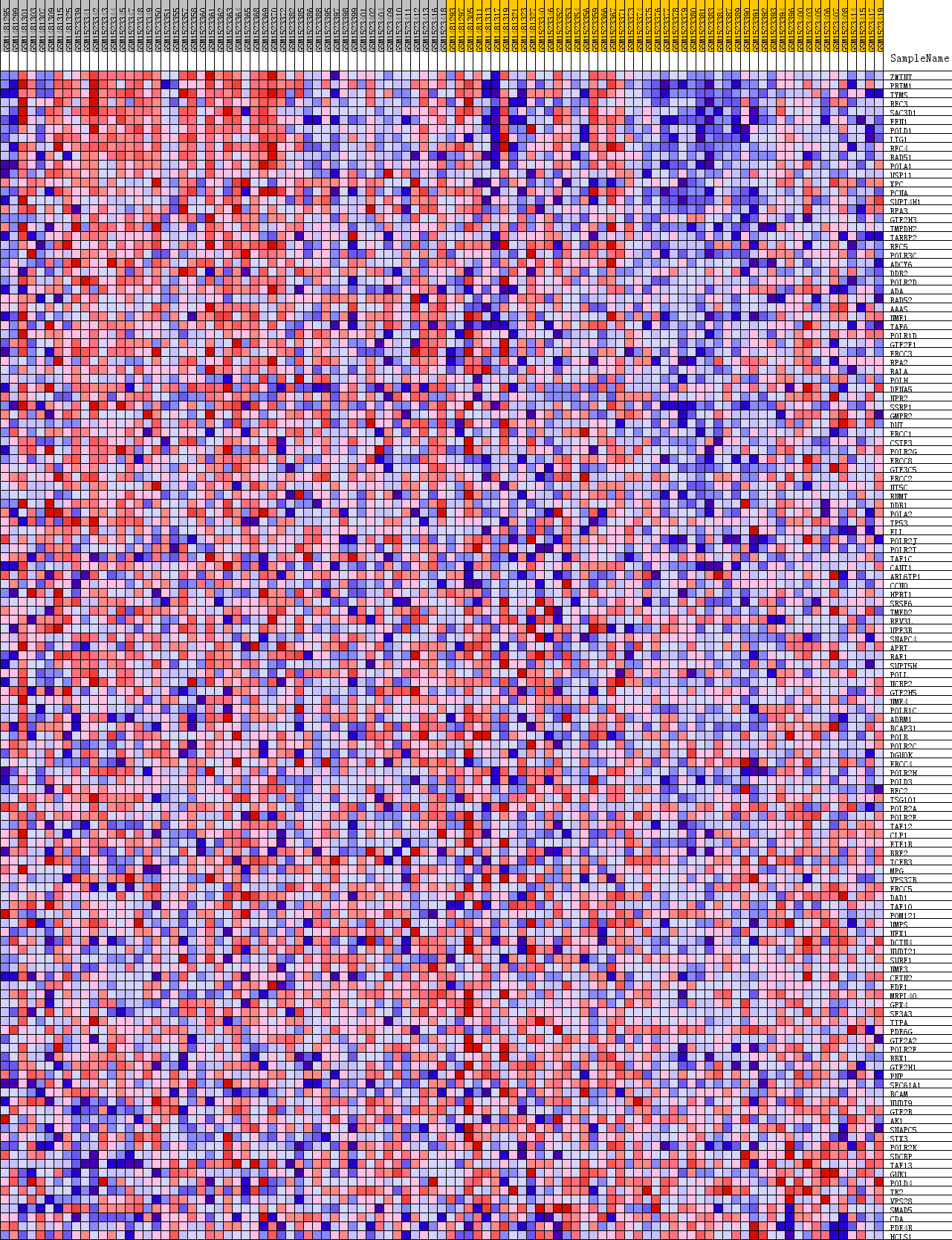

Supplement: Supplemental Information 2 — The data, heat map, Enrich the original picture and ES distribution plot of differentially enriched pathways. [file peerj-07-7816-s002.zip › GEO/h.all.v6.2.symbols.gmt/HALLMARK_DNA_REPAIR_4.png]

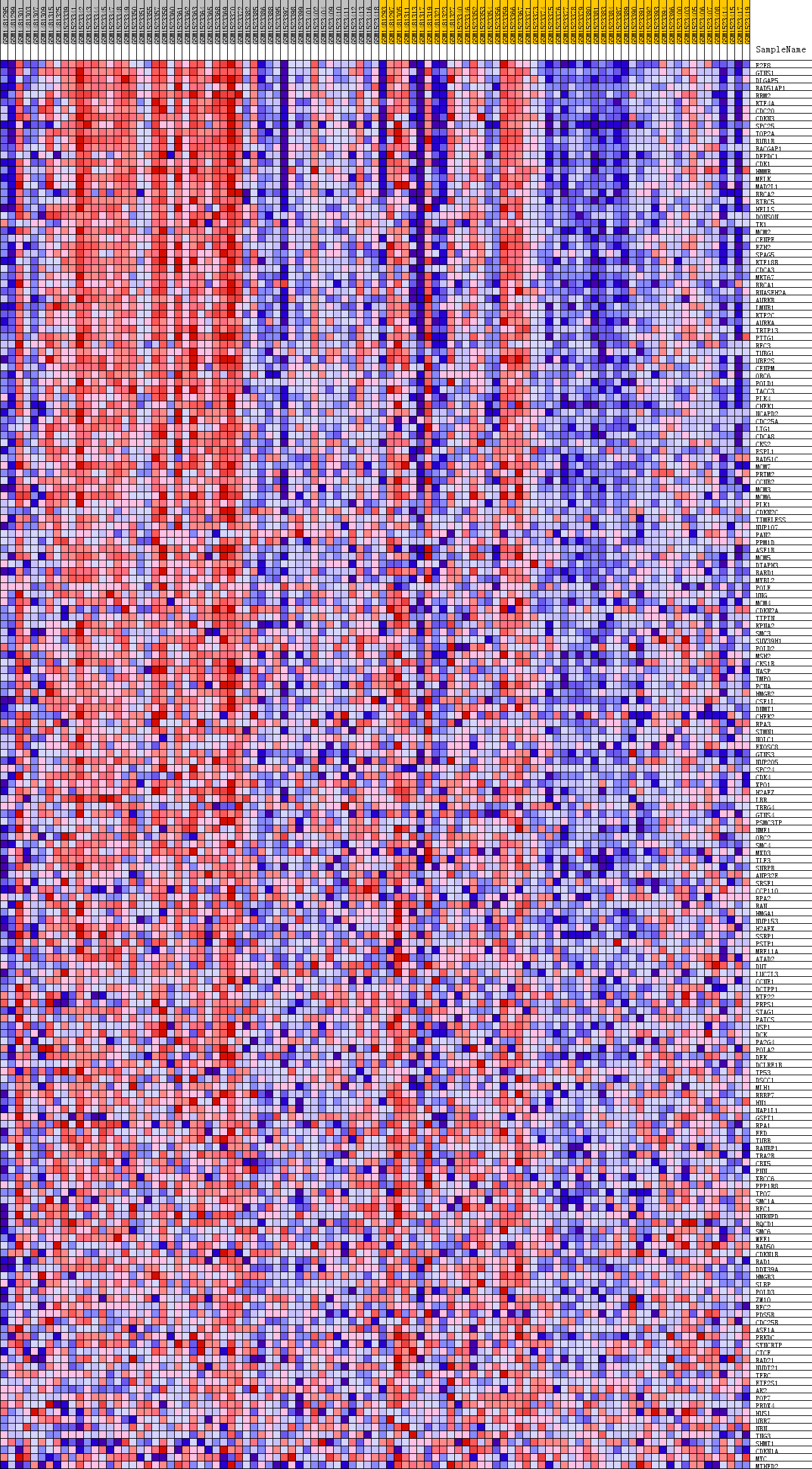

Supplement: Supplemental Information 2 — The data, heat map, Enrich the original picture and ES distribution plot of differentially enriched pathways. [file peerj-07-7816-s002.zip › GEO/h.all.v6.2.symbols.gmt/HALLMARK_E2F_TARGETS_16.png]

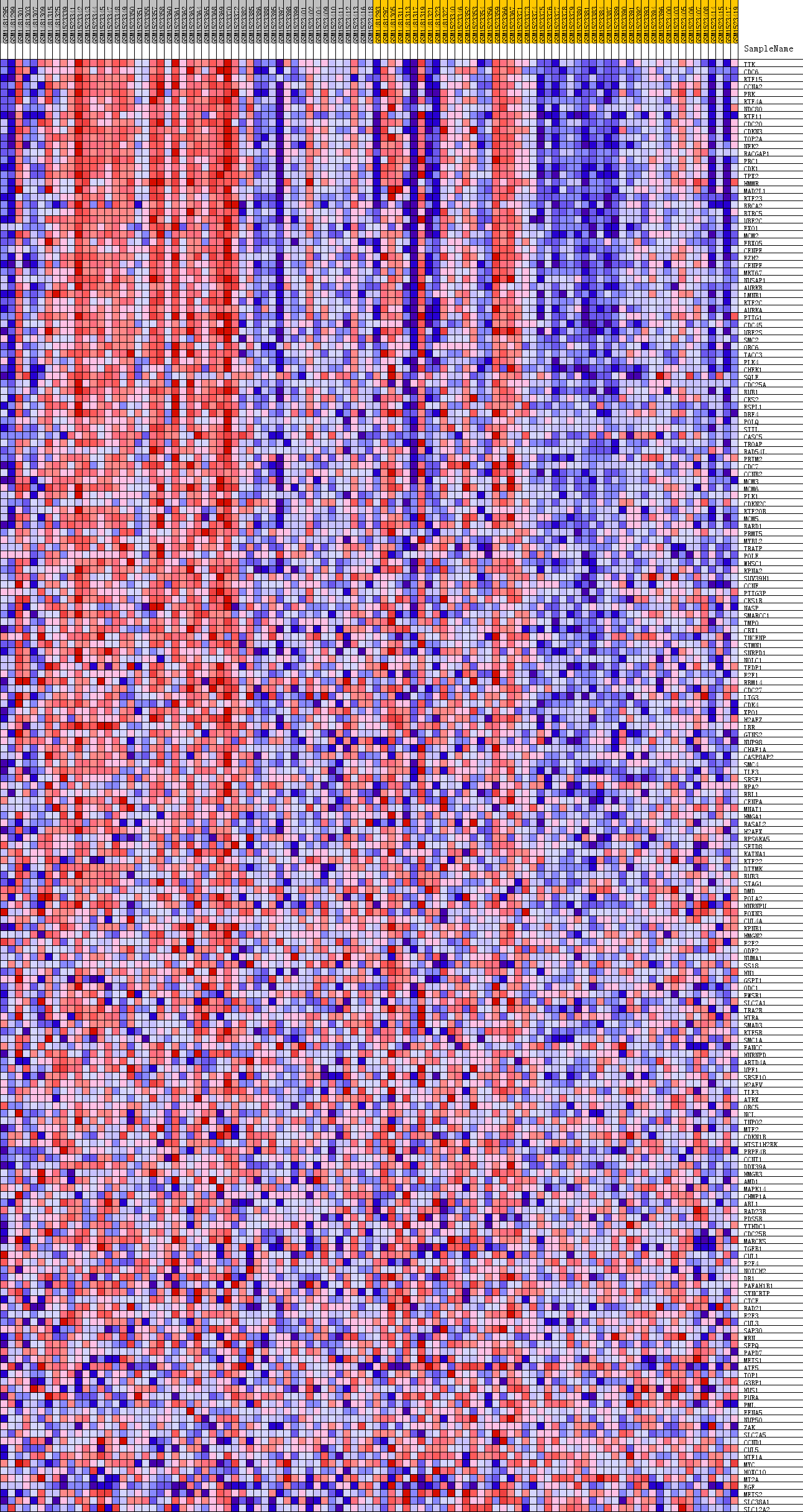

Supplement: Supplemental Information 2 — The data, heat map, Enrich the original picture and ES distribution plot of differentially enriched pathways. [file peerj-07-7816-s002.zip › GEO/h.all.v6.2.symbols.gmt/HALLMARK_G2M_CHECKPOINT_10.png]

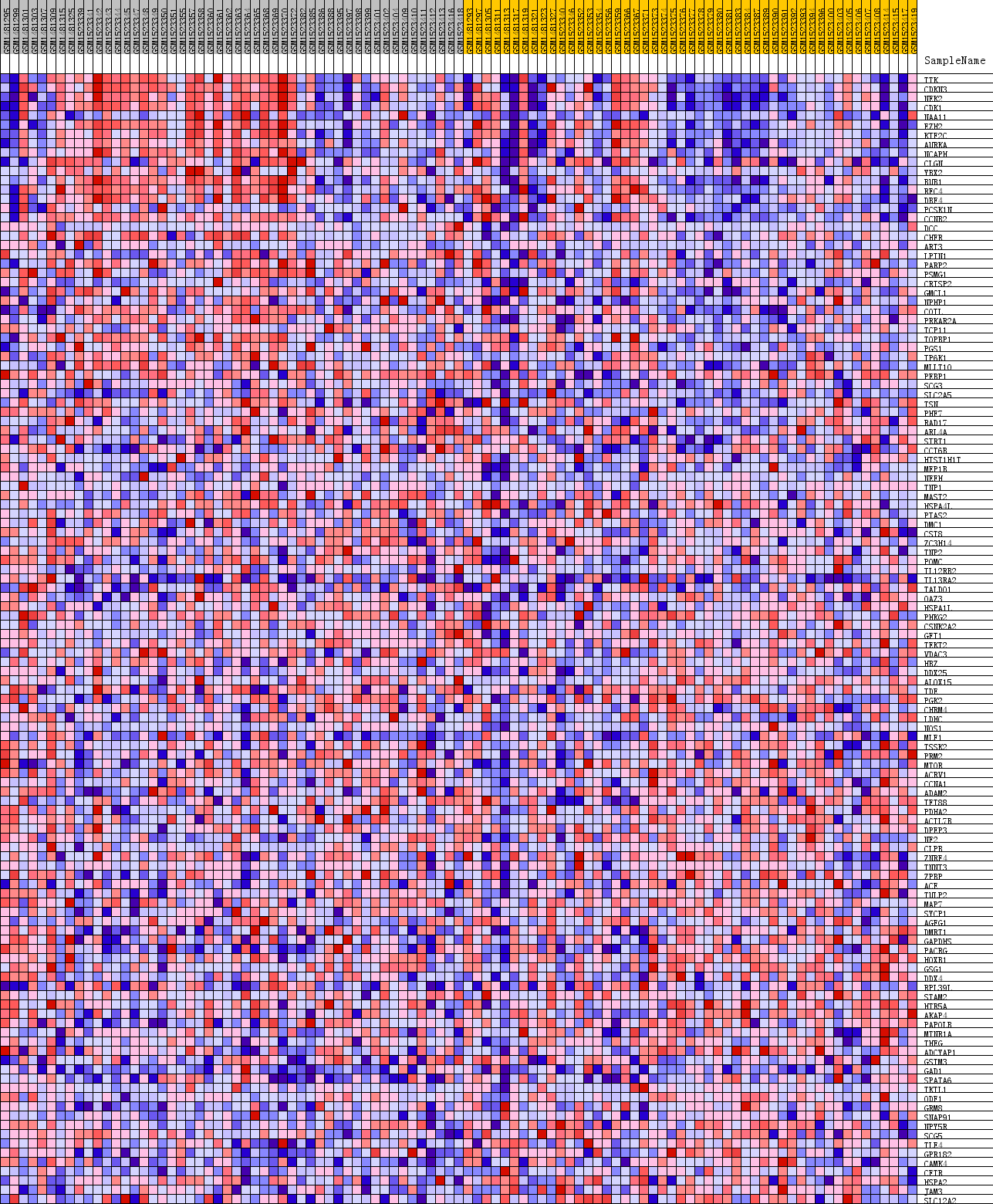

Supplement: Supplemental Information 2 — The data, heat map, Enrich the original picture and ES distribution plot of differentially enriched pathways. [file peerj-07-7816-s002.zip › GEO/h.all.v6.2.symbols.gmt/HALLMARK_SPERMATOGENESIS_13.png]

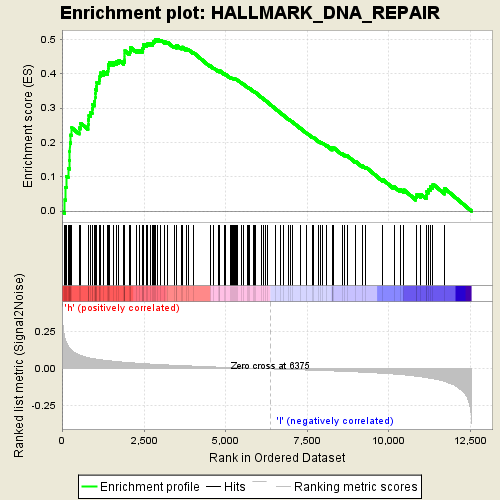

Supplement: Supplemental Information 2 — The data, heat map, Enrich the original picture and ES distribution plot of differentially enriched pathways. [file peerj-07-7816-s002.zip › GEO/h.all.v6.2.symbols.gmt/enplot_HALLMARK_DNA_REPAIR_3.png]

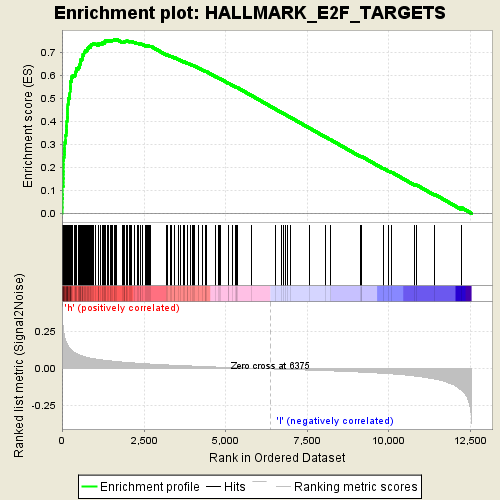

Supplement: Supplemental Information 2 — The data, heat map, Enrich the original picture and ES distribution plot of differentially enriched pathways. [file peerj-07-7816-s002.zip › GEO/h.all.v6.2.symbols.gmt/enplot_HALLMARK_E2F_TARGETS_15.png]

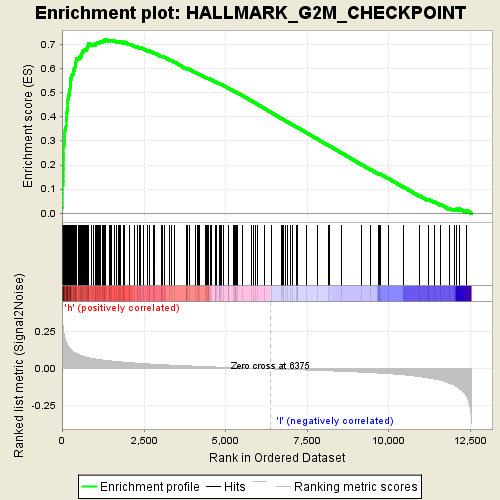

Supplement: Supplemental Information 2 — The data, heat map, Enrich the original picture and ES distribution plot of differentially enriched pathways. [file peerj-07-7816-s002.zip › GEO/h.all.v6.2.symbols.gmt/enplot_HALLMARK_G2M_CHECKPOINT_9.png]

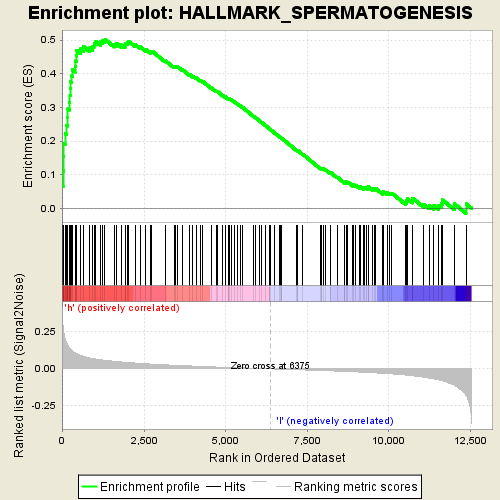

Supplement: Supplemental Information 2 — The data, heat map, Enrich the original picture and ES distribution plot of differentially enriched pathways. [file peerj-07-7816-s002.zip › GEO/h.all.v6.2.symbols.gmt/enplot_HALLMARK_SPERMATOGENESIS_12.png]

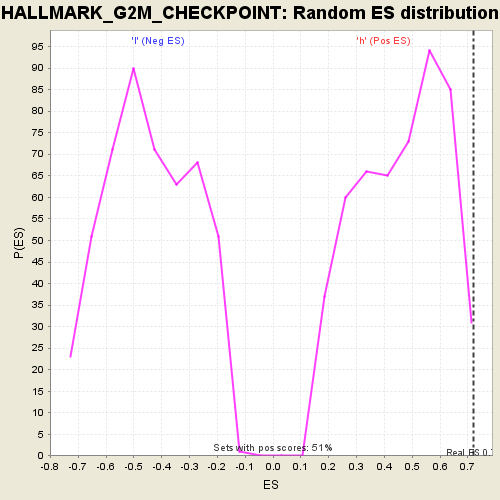

Supplement: Supplemental Information 2 — The data, heat map, Enrich the original picture and ES distribution plot of differentially enriched pathways. [file peerj-07-7816-s002.zip › GEO/h.all.v6.2.symbols.gmt/gset_rnd_es_dist_11.png]

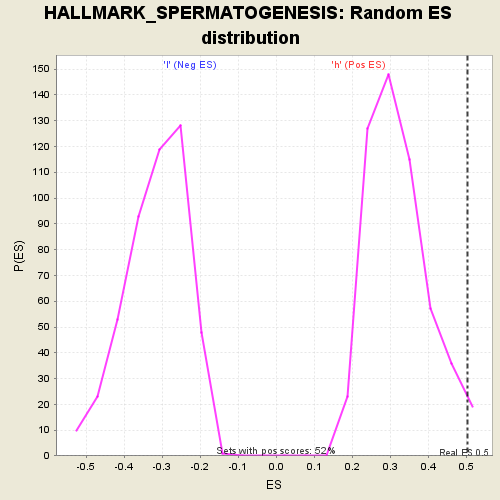

Supplement: Supplemental Information 2 — The data, heat map, Enrich the original picture and ES distribution plot of differentially enriched pathways. [file peerj-07-7816-s002.zip › GEO/h.all.v6.2.symbols.gmt/gset_rnd_es_dist_14.png]

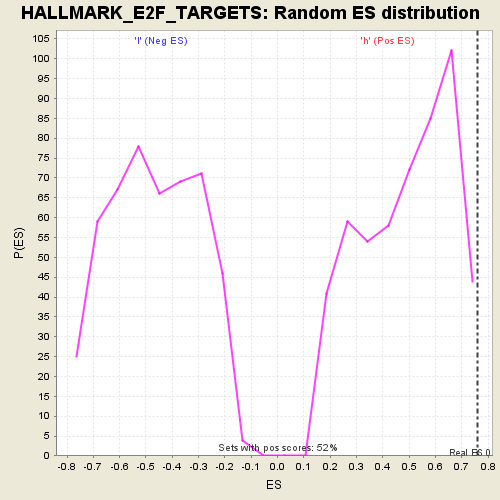

Supplement: Supplemental Information 2 — The data, heat map, Enrich the original picture and ES distribution plot of differentially enriched pathways. [file peerj-07-7816-s002.zip › GEO/h.all.v6.2.symbols.gmt/gset_rnd_es_dist_17.png]

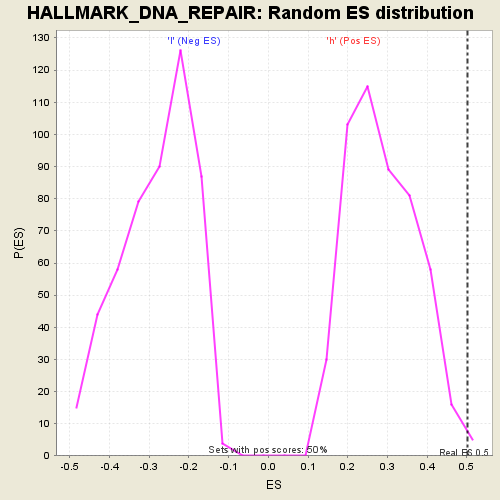

Supplement: Supplemental Information 2 — The data, heat map, Enrich the original picture and ES distribution plot of differentially enriched pathways. [file peerj-07-7816-s002.zip › GEO/h.all.v6.2.symbols.gmt/gset_rnd_es_dist_5.png]

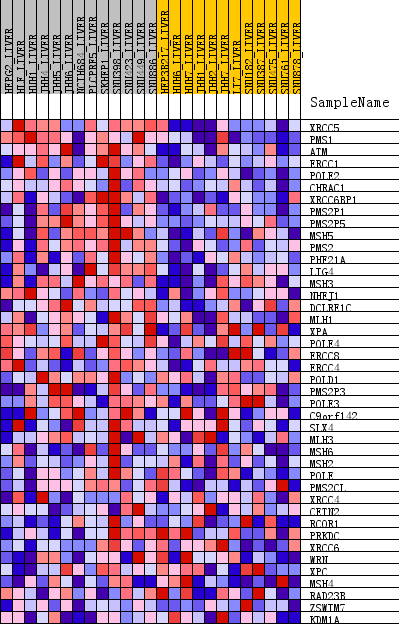

Supplement: Supplemental Information 3 — The data, heat map, Enrich the original picture and ES distribution plot of differentially enriched pathways. [file peerj-07-7816-s003.zip › CCLE/c5.all.v6.2.symbols.gmt/GO_DNA_REPAIR_COMPLEX_157.png]

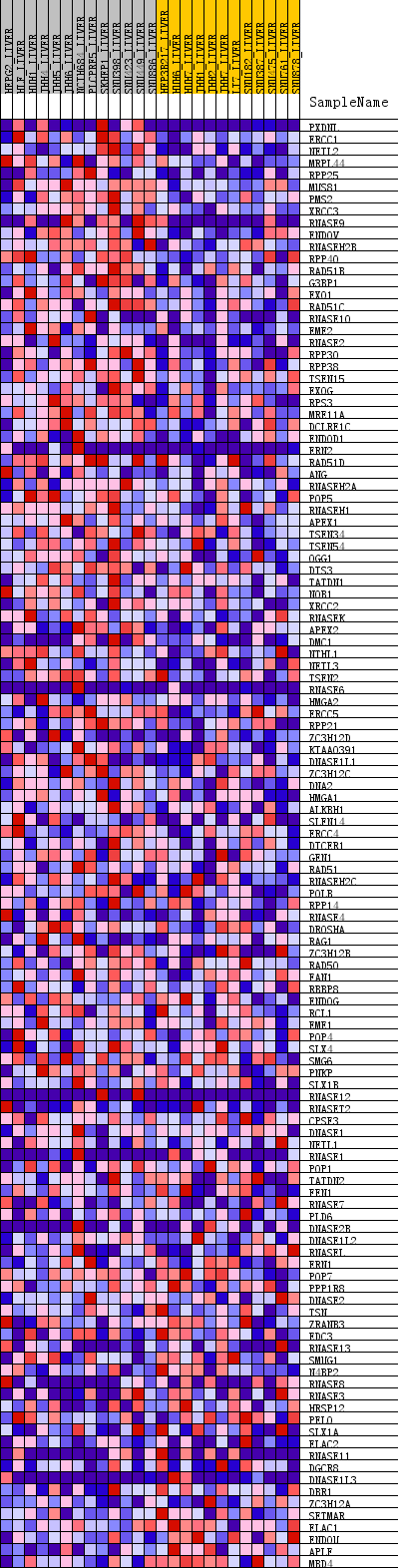

Supplement: Supplemental Information 3 — The data, heat map, Enrich the original picture and ES distribution plot of differentially enriched pathways. [file peerj-07-7816-s003.zip › CCLE/c5.all.v6.2.symbols.gmt/GO_ENDONUCLEASE_ACTIVITY_130.png]

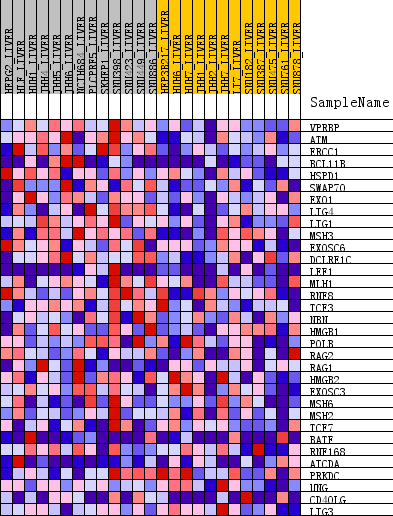

Supplement: Supplemental Information 3 — The data, heat map, Enrich the original picture and ES distribution plot of differentially enriched pathways. [file peerj-07-7816-s003.zip › CCLE/c5.all.v6.2.symbols.gmt/GO_SOMATIC_CELL_DNA_RECOMBINATION_166.png]

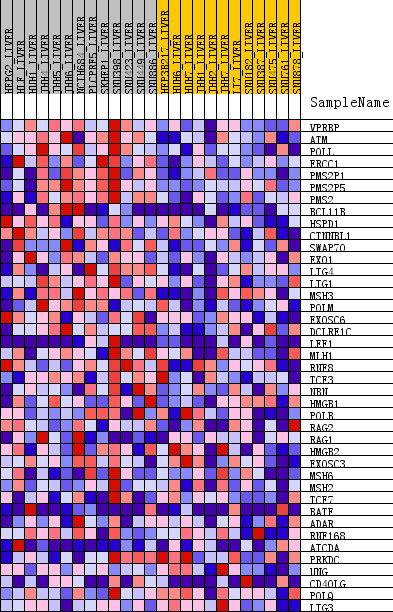

Supplement: Supplemental Information 3 — The data, heat map, Enrich the original picture and ES distribution plot of differentially enriched pathways. [file peerj-07-7816-s003.zip › CCLE/c5.all.v6.2.symbols.gmt/GO_SOMATIC_DIVERSIFICATION_OF_IMMUNE_RECEPTORS_58.png]

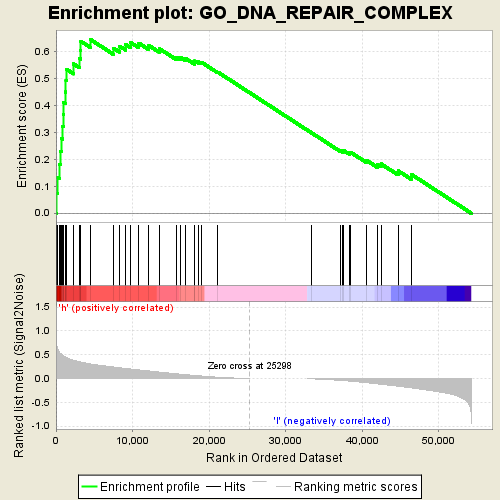

Supplement: Supplemental Information 3 — The data, heat map, Enrich the original picture and ES distribution plot of differentially enriched pathways. [file peerj-07-7816-s003.zip › CCLE/c5.all.v6.2.symbols.gmt/enplot_GO_DNA_REPAIR_COMPLEX_156.png]

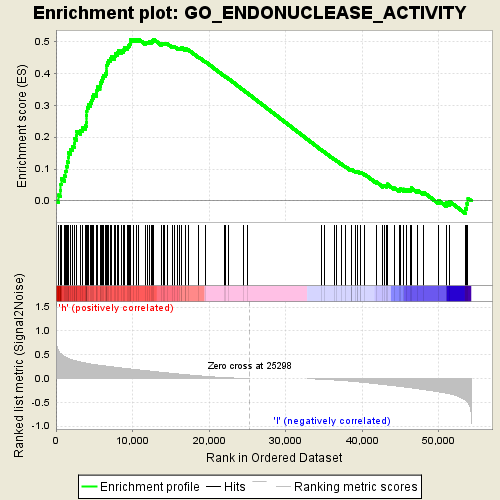

Supplement: Supplemental Information 3 — The data, heat map, Enrich the original picture and ES distribution plot of differentially enriched pathways. [file peerj-07-7816-s003.zip › CCLE/c5.all.v6.2.symbols.gmt/enplot_GO_ENDONUCLEASE_ACTIVITY_129.png]

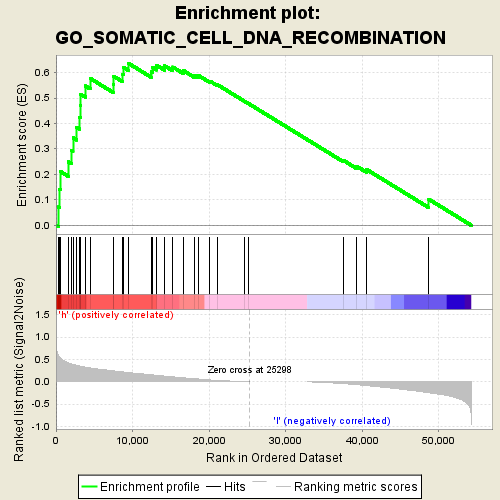

Supplement: Supplemental Information 3 — The data, heat map, Enrich the original picture and ES distribution plot of differentially enriched pathways. [file peerj-07-7816-s003.zip › CCLE/c5.all.v6.2.symbols.gmt/enplot_GO_SOMATIC_CELL_DNA_RECOMBINATION_165.png]

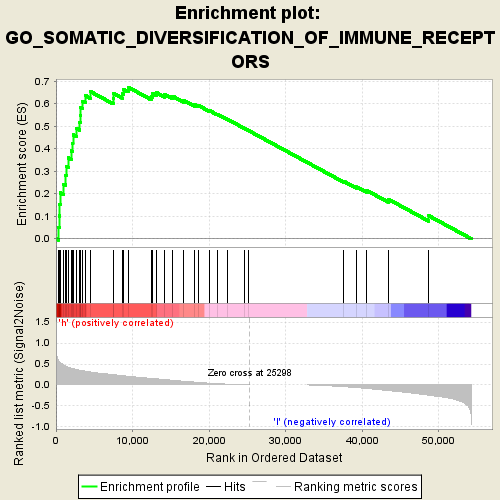

Supplement: Supplemental Information 3 — The data, heat map, Enrich the original picture and ES distribution plot of differentially enriched pathways. [file peerj-07-7816-s003.zip › CCLE/c5.all.v6.2.symbols.gmt/enplot_GO_SOMATIC_DIVERSIFICATION_OF_IMMUNE_RECEPTORS_57.png]

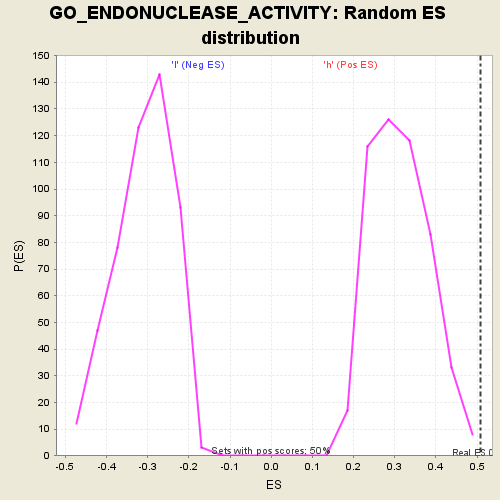

Supplement: Supplemental Information 3 — The data, heat map, Enrich the original picture and ES distribution plot of differentially enriched pathways. [file peerj-07-7816-s003.zip › CCLE/c5.all.v6.2.symbols.gmt/gset_rnd_es_dist_131.png]

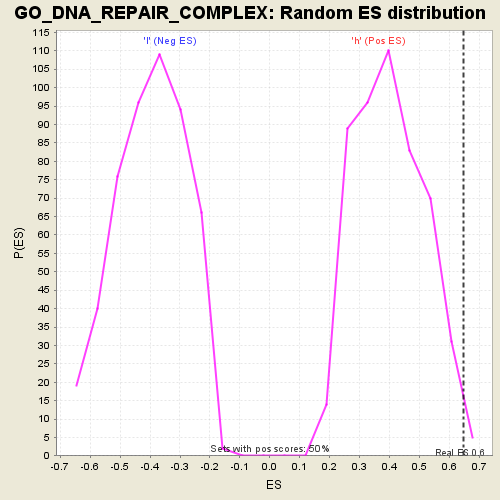

Supplement: Supplemental Information 3 — The data, heat map, Enrich the original picture and ES distribution plot of differentially enriched pathways. [file peerj-07-7816-s003.zip › CCLE/c5.all.v6.2.symbols.gmt/gset_rnd_es_dist_158.png]

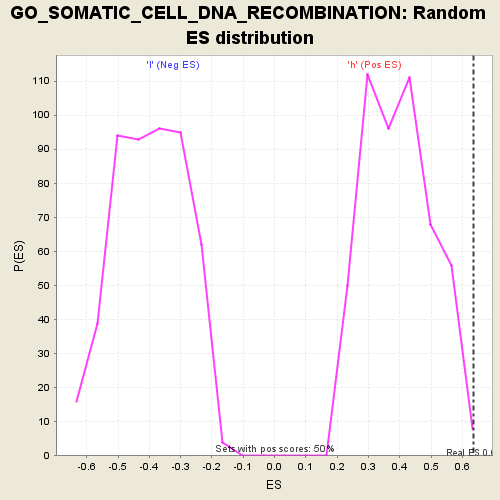

Supplement: Supplemental Information 3 — The data, heat map, Enrich the original picture and ES distribution plot of differentially enriched pathways. [file peerj-07-7816-s003.zip › CCLE/c5.all.v6.2.symbols.gmt/gset_rnd_es_dist_167.png]

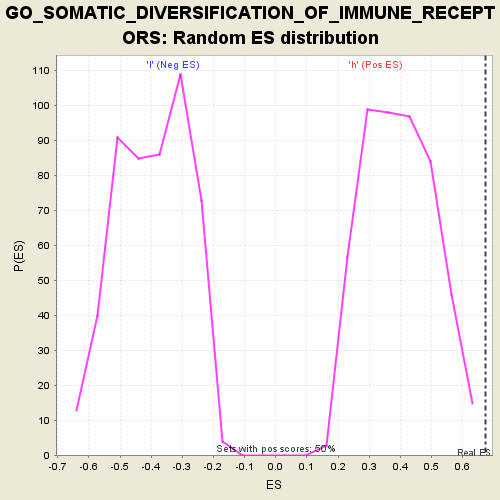

Supplement: Supplemental Information 3 — The data, heat map, Enrich the original picture and ES distribution plot of differentially enriched pathways. [file peerj-07-7816-s003.zip › CCLE/c5.all.v6.2.symbols.gmt/gset_rnd_es_dist_59.png]

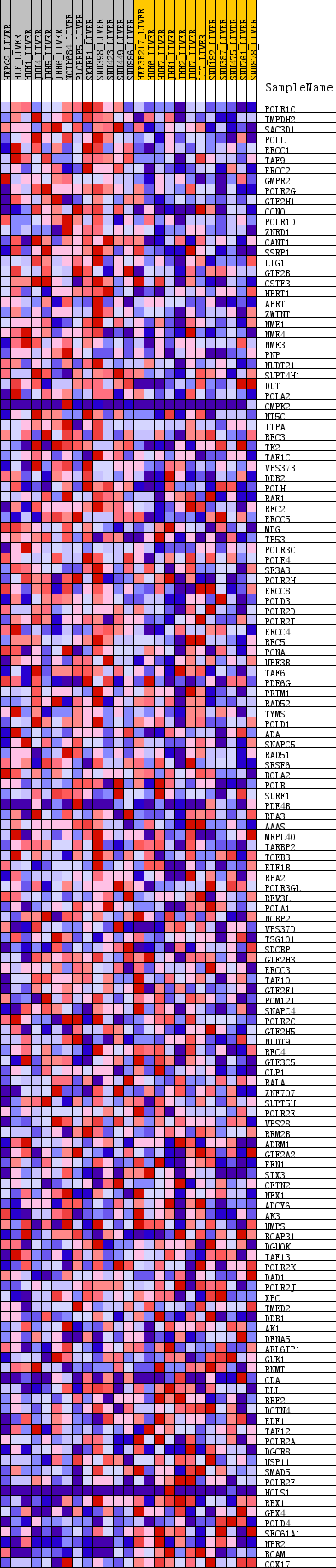

Supplement: Supplemental Information 3 — The data, heat map, Enrich the original picture and ES distribution plot of differentially enriched pathways. [file peerj-07-7816-s003.zip › CCLE/h.all.v6.2.symbols.gmt/HALLMARK_DNA_REPAIR_126.png]

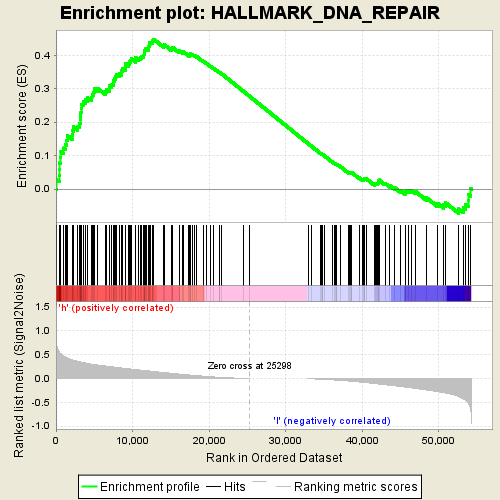

Supplement: Supplemental Information 3 — The data, heat map, Enrich the original picture and ES distribution plot of differentially enriched pathways. [file peerj-07-7816-s003.zip › CCLE/h.all.v6.2.symbols.gmt/enplot_HALLMARK_DNA_REPAIR_125.png]

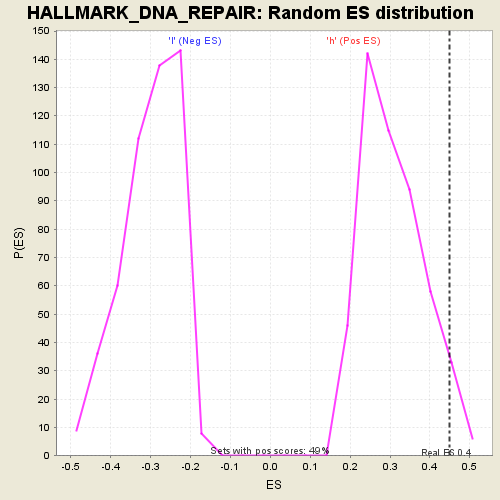

Supplement: Supplemental Information 3 — The data, heat map, Enrich the original picture and ES distribution plot of differentially enriched pathways. [file peerj-07-7816-s003.zip › CCLE/h.all.v6.2.symbols.gmt/gset_rnd_es_dist_127.png]
